# Comparative efficacy of interventions on nonalcoholic fatty liver disease (NAFLD): A PRISMA-compliant systematic review and network meta-analysis

**DOI:** 10.1097/MD.0000000000004529

**Published:** 2016-08-12

**Authors:** Ratree Sawangjit, Bunchai Chongmelaxme, Pochamana Phisalprapa, Surasak Saokaew, Ammarin Thakkinstian, Kris V. Kowdley, Nathorn Chaiyakunapruk

**Affiliations:** a Clinical Pharmacy Research Unit (CPRU), Department of Clinical Pharmacy, Faculty of Pharmacy, Mahasarakham University, Mahasarakham, Thailand; b School of Pharmacy, Monash University Malaysia, Selangor, Malaysia; c Center of Pharmaceutical Outcomes Research, Faculty of Pharmaceutical Sciences, Naresuan University, Phitsanulok; d Department of Medicine, Faculty of Medicine Siriraj Hospital, Mahidol University, Bangkok; e Center of Health Outcome Research and Therapeutic Safety, School of Pharmaceutical Sciences,University of Phayao, Phayao; f Section for Clinical Epidemiology and Biostatistics, Faculty of Medicine, Ramathibodi Hospital, Mahidol University, Bangkok, Thailand; g Liver Care Network and Organ Care Research, Swedish Medical Center, Seattle; h School of Pharmacy, University of Wisconsin, Madison, WI; i School of Population Health, University of Queensland, Brisbane, QLD, Australia.

**Keywords:** fibrosis, GRADE, histological outcome, NAFLD, NASH, network meta-analysis

## Abstract

**Background:**

The prevalence of nonalcoholic fatty liver disease (NAFLD) has significantly increased over the last decades. Despite existence of several interventions, there remains unclear which interventions work the best.

**Methods:**

A systematic review and network meta-analysis of randomized trials comparing efficacy of all treatment options in NAFLD were performed to determine comparative efficacy and safety of interventions in the management of NAFLD. Several electronic databases were searched up to Nov 15, 2015. Outcomes include liver histological outcomes (i.e., fibrosis), all-cause mortality, cirrhosis, and safety. A network meta-analysis was applied to estimate pooled risk ratios (RR). Quality of evidence was assessed using GRADE criteria.

**Results:**

A total of 44 studies (n = 3802) were eligible. When compared with placebo, obeticholic acid (OCA) was the only intervention that significantly improved fibrosis with RR (95% CI) of 1.91 (1.15, 3.16), while pentoxyfylline (PTX) demonstrated improved fibrosis without statistical significance with RR (95% CI) of 2.27 (0.81, 6.36). Only thiazolidinedione (TZD) and vitamin E use resulted in significant increase in resolution of NASH, while OCA, TZD, and vitamin E significantly improved other outcomes including NAS, steatosis, ballooning, and inflammation outcomes. Quality of evidence varied from very low (i.e., metformin, PTX on mean change of ballooning grade) to high (OCA, TZD, vitamin E on improving histological outcomes). Limitations of this study were lack of relevant long-term outcomes (e.g., cirrhosis, death, safety), possible small study effect, and few head-to-head studies.

**Conclusions:**

Our study suggests potential efficacy of OCA, TZD, and vitamin E in improving histologic endpoints in NAFLD. These findings are however based on a small number of studies. Additional studies are awaited to strengthen this network meta-analysis.

## Abstract 2

Supplemental Digital Content is available in the text

## 1 Introduction

Nonalcoholic fatty liver disease (NAFLD) is defined as excessive fat (i.e., triglyceride) accumulation in the liver without secondary hepatic fat accumulation such as significant alcohol consumption, use of steatogenic medication, or hereditary disorders. ^[1]^ It is histologically categorized into nonalcoholic fatty liver (NAFL) and nonalcoholic steatohepatitis (NASH). NAFL is defined as the presence of hepatic steatosis without hepatocellular injury, whereas NASH is characterized by NAFL with hepatocellular ballooning injury with or without fibrosis. ^[1]^ NAFLD is now the most common cause of chronic liver disease worldwide ^[2]^ and affects 15% to 30% of the general population but is more prevalent (about 50%–90%) in patients with diabetes, metabolic syndrome, and severe obesity.[ ^1^ ^3^] Current evidence suggests that 68% of adults in the United States are overweight, estimating that 75 to 100 million individuals may have NAFLD. ^[4]^ NAFLD is associated both with an increased risk of liver-related complications, for examples, liver fibrosis (41%), cirrhosis (20%–25%), end-stage liver disease (5.4%), and cardiovascular disease (CVD).[ ^4^ ^5^]

The pathophysiologic mechanisms in NAFLD remain incompletely understood; therapy is therefore empiric and has mainly emphasized treatment of the associated conditions (e.g., diabetes, obesity, hyperlipidemia) including lifestyle modifications (e.g., weight loss, diet, and exercise). Both nonpharmacological and pharmacological interventions seem to play important roles ^[6]^ and have been investigated, [^7^ ^8^ ^9^ ^10^ ^11^ ^12^ ^13^] but it remains unclear which interventions are the most efficacious for NAFLD managements.

A traditional pair-wise meta-analysis could answer which treatment is better than placebo, but not for comparison of multiple treatment options.[ ^14^ ^15^] Applying a network meta-analysis by borrowing data from common comparators may lead us to indirectly compare multiple interventions and thus answer this question. Therefore, we conducted a systematic review and a network meta-analysis to compare the efficacy and safety of multiple interventions in the management of NAFLD.

## 2 Methods

This systematic review was conducted according to the Preferred Reporting Items for Systematic Reviews and Meta-Analyses (PRISMA) Extension Statement for Reporting of Systematic Reviews Incorporating Network Meta-analyses of Health Care Interventions, ^[16]^ and was conducted following a priori established protocol in Prospero (CRD42015025051). ^[17]^ We used GRADE criteria for network meta-analysis to assess quality of evidence.[ ^18^ ^19^]

### 2.1 Data sources and searches

We identified randomized controlled trials (RCTs) published up to November 15, 2015 and compared different interventions for NAFLD from the following databases: PubMed, the Cochrane Library Central Register of Controlled Trials (CENTRAL), Embase, CINAHL, Web of Science, Scopus, ClinicalTrials.gov, and WHO registry. We developed and modified search algorithms properly for each database by combining relevant search terms following Cochrane for systematic reviews of RCTs suggestions. ^[20]^ Uses of search strategies were clearly described in Appendix Table 1. Reference lists of relevant studies were also screened. Two investigators (RS, BC) independently reviewed the titles and abstracts and the full articles were evaluated if a decision could not be made for selections. Disagreements were resolved by discussion with NC.

### 2.2 Study selection

RCTs were included if they met the following inclusion criteria: biopsy-proven NAFLD; any type of nonpharmacological and pharmacological interventions, single or combined interventions as sole, or adjunct therapy; a placebo or active comparator; and use of biopsy-based histological outcomes. Studies were excluded if insufficient data, or Traditional Chinese Medicine (TCM) or probiotics interventions.

### 2.3 Data extraction and quality assessment

Data were independently extracted by 2 investigators (RS, BC) using the standardized data extraction forms. These included patient characteristics, time to follow-up, histological characteristics, types of interventions, and outcomes (definitions and measurements). The risk of bias (ROB) was assessed using Cochrane risk of bias tool, ^[20]^ which consisted of 6 items (i.e., sequence generation, allocation concealment, blinding, incomplete outcome data, selective outcome reporting, and other biases). Each item was rated as high, low, or unclear.

### 2.4 Type of interventions

Types of interventions were categorized into 9 main groups and combinations of them including antioxidants (Antiox), metformin (Met), pentoxifyline (PTX), polyunsaturated fatty acid (PUFA), thiazolidinedione (TZD), ursodeoxycholic acid (UDCA), vitamin E plus C (vitamin E/vitamin C), weight/lipid control (pharmacology and nonpharmacology), others (obeticholic acid; OCA, Metadoxine, Betaine, Valsartan, and Tryptophan/phospholipid).

### 2.5 Outcomes of interest

The primary outcomes were improvement of fibrosis, death either overall or related to liver and cardiovascular disease deaths, and cirrhosis. Secondary outcomes were improvement of ballooning degeneration, steatosis, lobular inflammation, and NAS, mean changes in NAS, ballooning, steatosis, and lobular inflammation, adverse effects. If outcomes were repeatedly assessed, we considered only at the end of the study. The denominator (the number of patients at risk) was based on intention-to-treat analysis. The patients without follow-up biopsy (or with lack of information on follow-up histological findings) were defined as treatment failures.

### 2.6 Quality of evidence

GRADEpro GDT software online version (http://www.guidelinedevelopment.org/ (access Sep 2015)) was used to evaluate quality of evidence from direct and network meta-analysis. Quality of evidence was categorized into 4 levels including, high, moderate, low, and very low.[ ^18^ ^19^] Details on grading are provided in Appendix Table 2. The quality of evidence for each pooled outcome was graded based on 5 domains including risk of bias, inconsistency, indirectness, imprecision, and publication bias.

### 2.7 Data synthesis and analysis

A pairwise meta-analysis with a random-effects model ^[21]^ was used to estimate treatment effects, pooled risk ratios (RR), or weighted mean differences (WMD) along with 95% confidence intervals (CI) for dichotomous and continuous outcomes, respectively. Heterogeneity was assessed using *χ* ^*2*^ test and *I* ^*2*^.[ ^20^ ^22^] If there was evidence of heterogeneity, we attempted to explore its sources (i.e., risk of bias criteria, study characteristics, and patient characteristics) by performing subgroup analyses.

A network meta-analysis was applied to indirectly compare intervention effects for all NAFLD managements with the following steps: coefficients (i.e., lnRR) along with variance–covariance of comparisons were estimated for each study using placebo or common interventions as comparators. Then, these lnRRs were pooled across studies using a multivariate meta-analysis with restricted maximum likelihood function. Between-study variance and covariance were taken into account using exchangeable method. Inconsistency assumption (i.e., agreement of direct and indirect effects) was checked by estimating inconsistency factor (IF) using design-by-treatment and node splitting technique model. ^[23]^ In addition, the IF was tested using *Z* test indicating inconsistency if the IF is significantly different from 0. The surface under the cumulative ranking curve (SUCRA) was performed based on Bayesian approach to measure the ranking and the uncertainty. A probability of being best intervention was also estimated.

An adjusted funnel plot was constructed to examine small study effects. ^[24]^ A sensitivity analysis was performed accordingly based on size of included RCTs. ^[25]^ We also performed prespecified subgroup/sensitivity analyses according to patient characteristics (i.e., age, obesity, DM, dosage and characteristics of interventions, period of follow-up (i.e., 6 months, 12 months, >12 months), procedure of staging outcome (i.e., NASH CRN ^[26]^ or Brunt method) ^[27]^ and study characteristics (i.e., study design, sample size, quality of study). All analyses were performed using STATA 14.0 (Stata Corp, College Station, TX). A *P* value ≤0.05 was considered statistically significant.

### 2.8 Ethical approval

Ethical approval was not required for this study. It is a systematic review and meta-analysis which was not affected patients directly.

## 3 Results

A total of 3216 relevant articles were identified (Appendix Figure 1). After duplication removal, 1896 articles were eligible for screening based on titles and abstracts, 1774 articles were excluded, leaving 122 articles for review. Finally, 44 RCTs involving a total of 3802 patients were included in our study.

### 3.1 Characteristics of included studies and quality of studies

Uses of intervention and comparator of 44 included studies[ ^8^ ^28^ ^29^ ^30^ ^31^ ^32^ ^33^ ^34^ ^35^ ^36^ ^37^ ^38^ ^39^ ^40^ ^41^ ^42^ ^43^ ^44^ ^45^ ^46^ ^47^ ^48^ ^49^ ^50^ ^51^ ^52^ ^53^ ^54^ ^55^ ^56^ ^57^ ^58^ ^59^ ^60^ ^61^ ^62^ ^63^ ^64^ ^65^ ^66^ ^67^ ^68^ ^69^ ^70^] are summarized in Table 1 . Among them, 35, 8, and 1 study were 2-arm,[ ^8^ ^28^ ^29^ ^30^ ^31^ ^33^ ^35^ ^37^ ^38^ ^39^ ^42^ ^43^ ^44^ ^45^ ^46^ ^47^ ^48^ ^49^ ^50^ ^51^ ^52^ ^54^ ^55^ ^56^ ^59^ ^60^ ^61^ ^62^ ^63^ ^64^ ^66^ ^67^ ^68^ ^69^ ^70^] 3-arm,[ ^32^ ^34^ ^40^ ^41^ ^53^ ^57^ ^58^ ^65^] and 4-arm ^[36]^ RCTs, respectively. Among 2-arm RCTs, 31 and 4 RCTs were placebo and active controls, respectively. Among 31 placebo controls, following various interventions were used: 8 studies for weight/lipid controls,[ ^42^ ^43^ ^48^ ^49^ ^50^ ^54^ ^64^ ^70^] 3 studies for TZD,[ ^29^ ^31^ ^55^] and PTX,[ ^56^ ^67^ ^69^] 4 studies for Met,[ ^39^ ^62^ ^63^ ^66^] and PUFA,[ ^30^ ^33^ ^45^ ^52^] 2 studies for antioxidants,[ ^47^ ^68^] UDCA,[ ^44^ ^46^] vitamin E/vitamin C,[ ^38^ ^51^] and other groups including betaine, ^[28]^ metadoxine, ^[61]^ and OCA, ^[8]^ respectively. Among 4 trials of 2-arm RTCs with active controls, their interventions and active comparators were as follows: telmisartan versus valsartan, ^[35]^ vitamin E versus bicyclol, ^[37]^ vitamin E versus vitamin E/TZD, ^[59]^ and TZD versus PTX. ^[60]^

**Table 1:**
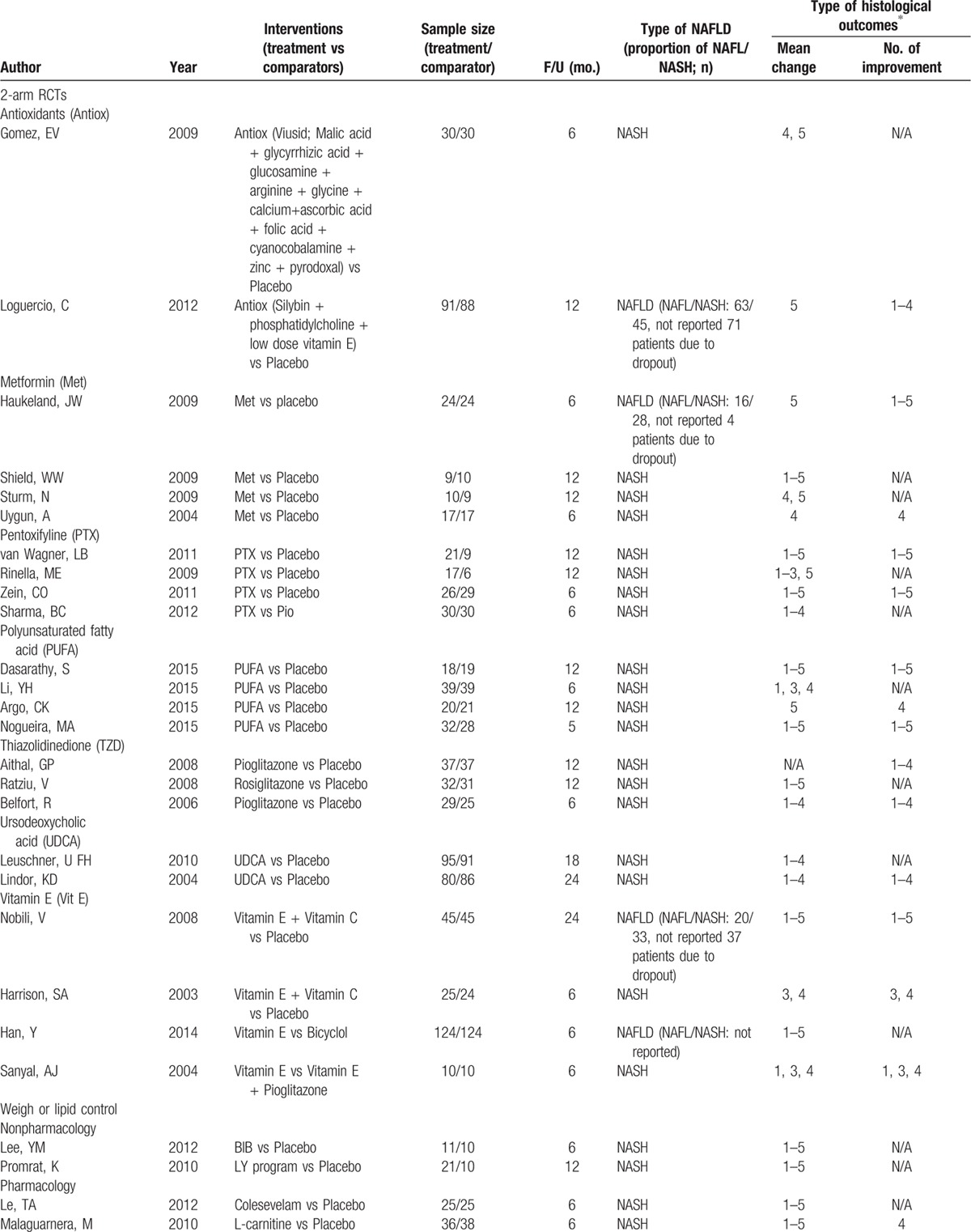
Characteristic of studies included in the network meta-analysis.

**Table 1 (Continued):**
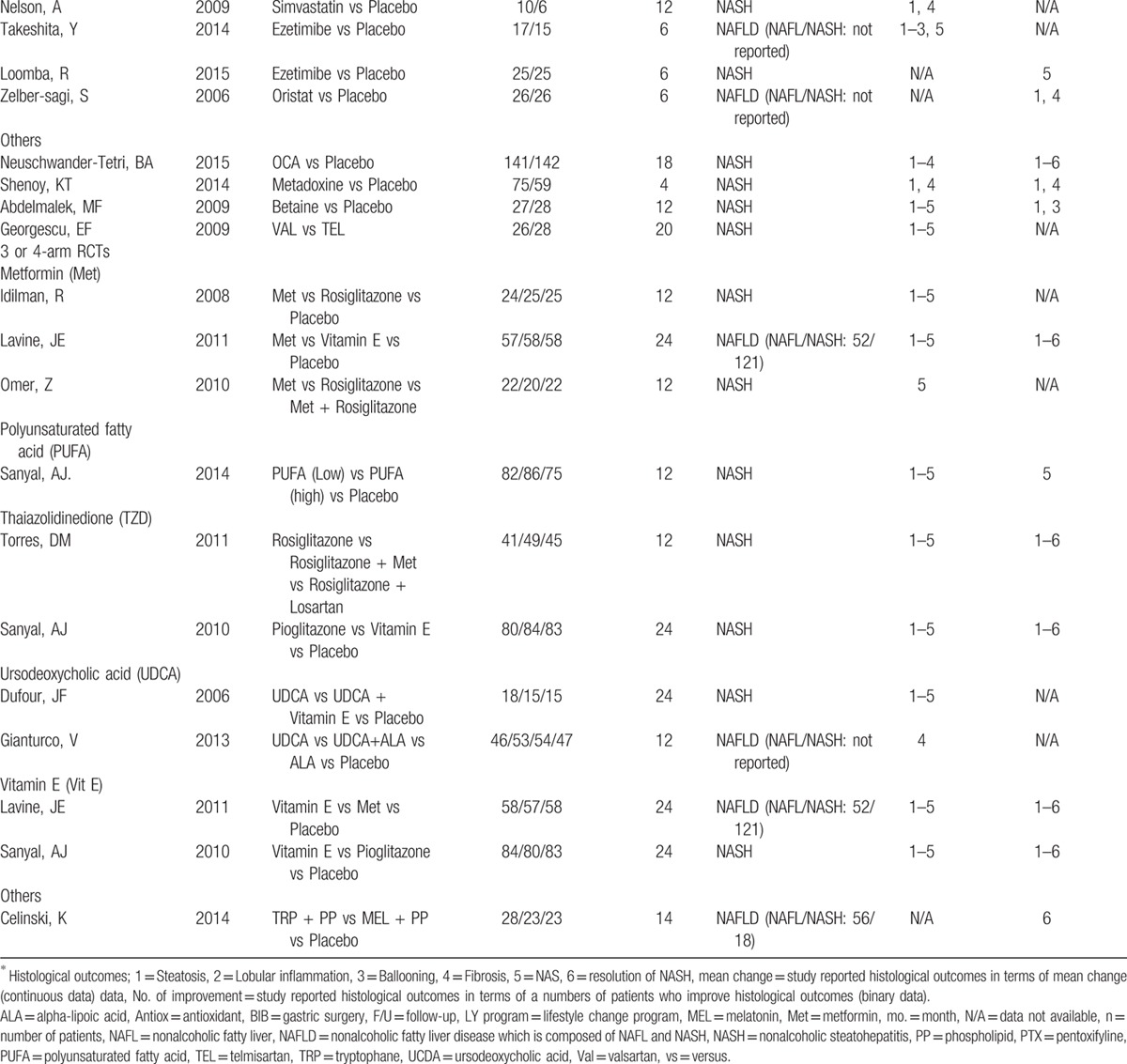
Characteristic of studies included in the network meta-analysis.

Among 3-arm RCTs, comparators were placebo in 6 RCTs,[ ^32^ ^34^ ^40^ ^41^ ^57^ ^58^] and active controls in 2 RCTs (Met vs TZD vs Met/TZD, ^[53]^ and TZD vs TZD/Met vs TZD/losartan). ^[65]^ The interventions for these 6 RCTs were Met or TZD, ^[40]^ PUFA (low/high dose), ^[57]^ UDCA or UDCA/vitamin E, ^[34]^ vitamin E or Met, ^[41]^ vitamin E or TZD, ^[58]^ and tryptophan/phospholipid or melatonin/phospholipid. ^[32]^ Only 1 was 4-arm trial, which compared effects of UDCA, Alpha-lipoic acid (ALA), UDCA/ALA, and placebo. ^[36]^

A total sample size in the 44 RCTs ranged from 16 to 283 patients (median = 58), duration of study/time at outcome measurement ranged from 4 to 24 months (median = 12). Most RCTs were conducted in the United States (N = 20), followed by European (EU) (N = 18), and Asian countries (N = 5). Most RCTs (34/44 studies) studied NASH patients and some (10/44) were in NAFLD patients. All RCTs studied in adults (aged 33–62 years, median = 48), except 2 RCTs studied in children.[ ^41^ ^51^] Patients with obesity were included in most RCTs (37/44), only diabetes in a few RCTs,[ ^31^ ^33^] and diabetes (ranged 9% to 53%) mixed with general in 20/44 RCTs.

Quality of included studies based on Cochrane risk of bias (ROB) tool was assessed, which suggested that 39%, 34%, and 27% of studies were low, unclear, and high quality, respectively (Appendix Figure 2). Most domains had at least 75% low risk of bias, except blinding had only 53%.

**Figure 1:**
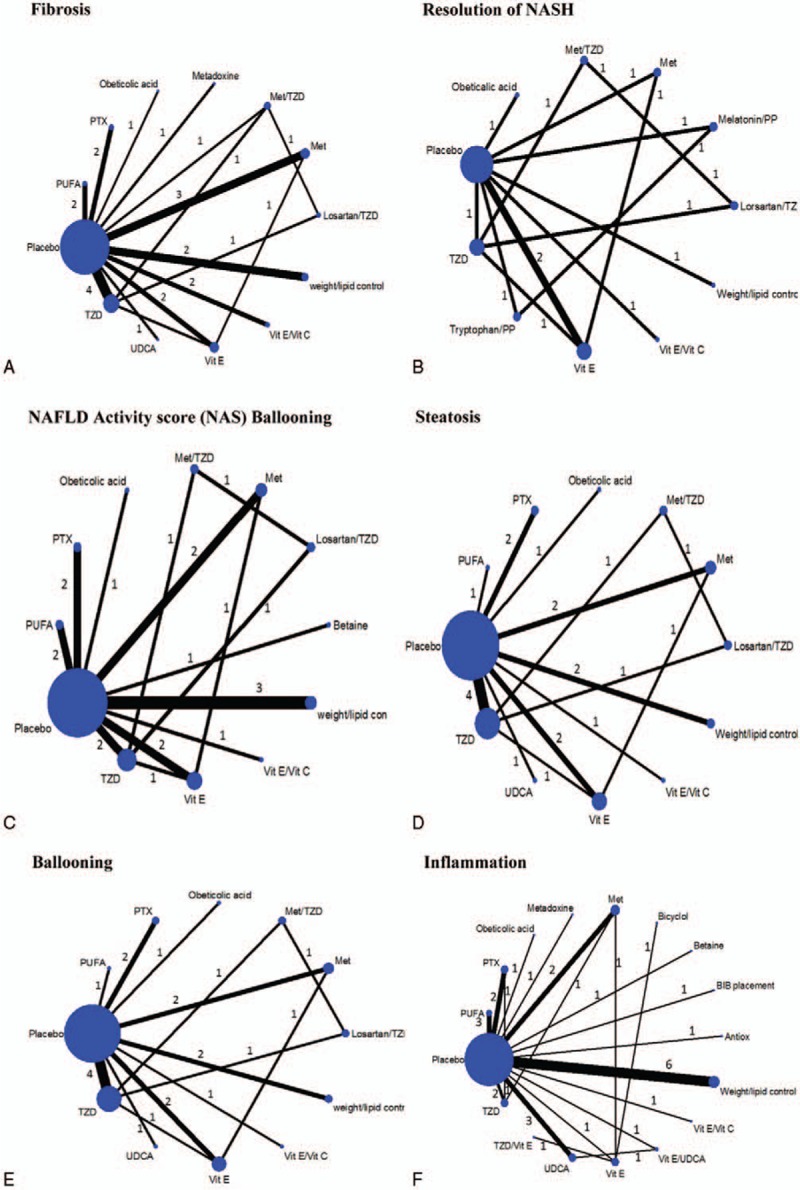
Network map of binary outcomes for improvement of histological outcomes. A, Fibrosis. B, Resolution of NASH (nonalcoholic steatohepatitis). C, NAFLD activity score (NAS). D, Steatosis. E, Ballooning degeneration. F, Lobular inflammation. Met = metformin, PP = phospholipid, PTX = pentoxifyline, PUFA = polyunsaturated fatty acid, TZD = thiazolidinedione, UCDA = ursodeoxycholic acid, Vit C = vitamin C, Vit E = vitamin E.

The primary outcomes were improvement of fibrosis, death (overall death, death related to liver, and cardiovascular diseases) and cirrhosis. A total of 21 RCTs reported improvement of fibrosis, 4 reported deaths but none reported reverse or development of cirrhosis during study period (Tables 1 and 2 ). Among 44 RCTs, NASH CRN's technique was used for grading histological outcomes by a blinded histologist in most studies (80%; 35/44). Figure 1A–F presents network map of all interventions for network meta-analysis in improvement of fibrosis, resolution of NASH, improvement of NAFLD activity score (NAS), steatosis, ballooning degeneration, and lobular inflammation, respectively.

**Table 2:**
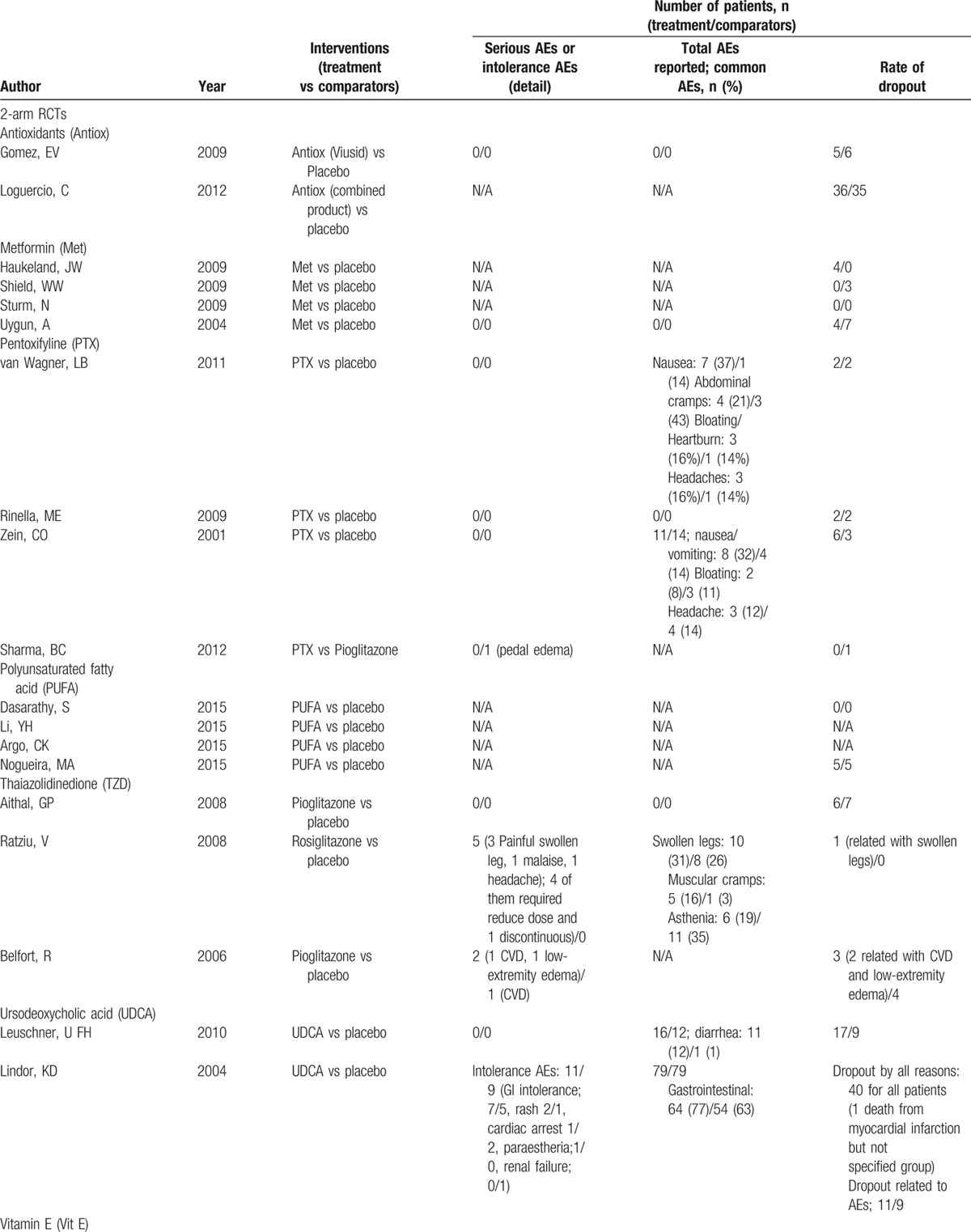
Adverse events and rates of participant dropout of all include studies.

**Table 2 (Continued):**
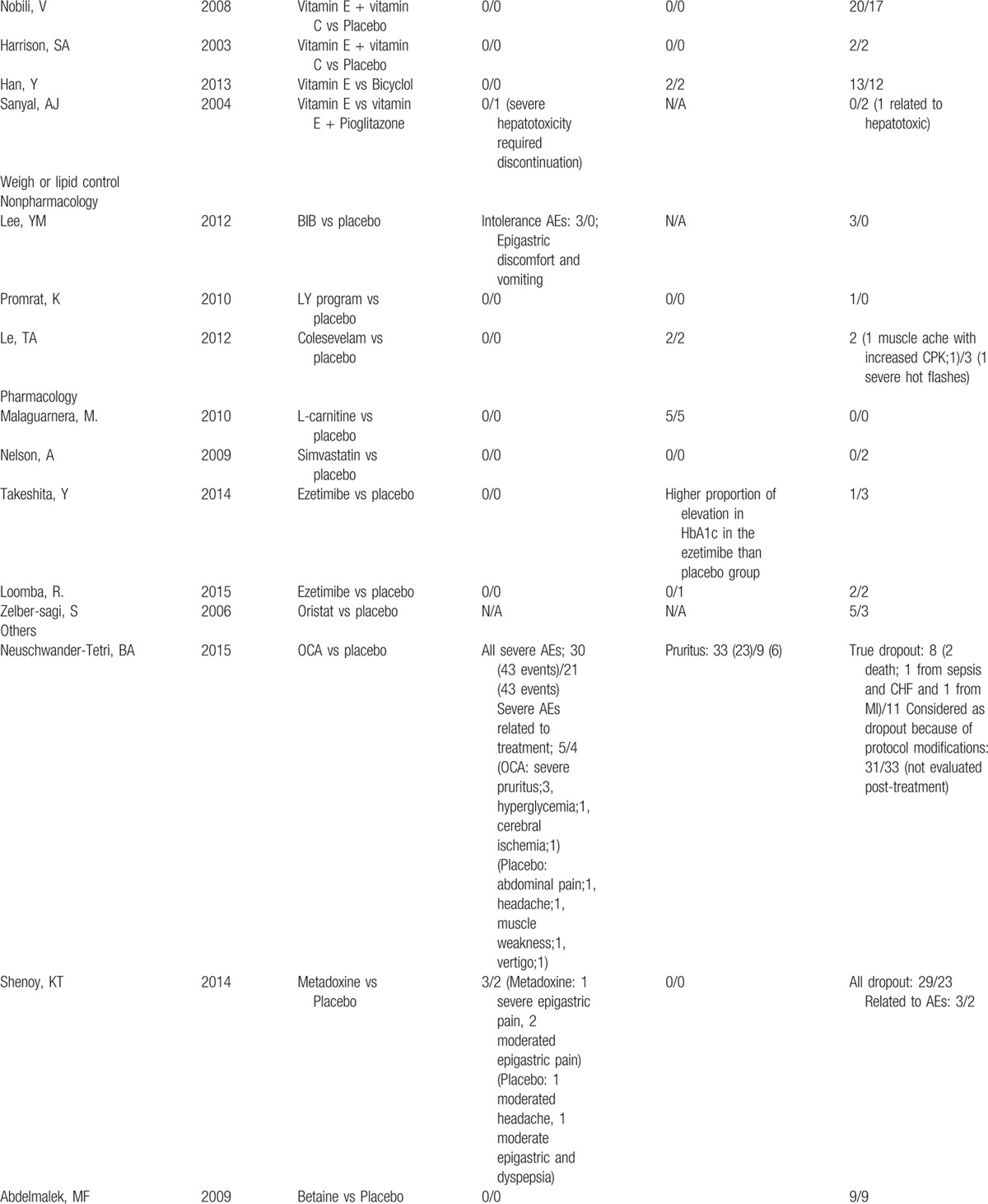
Adverse events and rates of participant dropout of all include studies.

**Table 2 (Continued):**
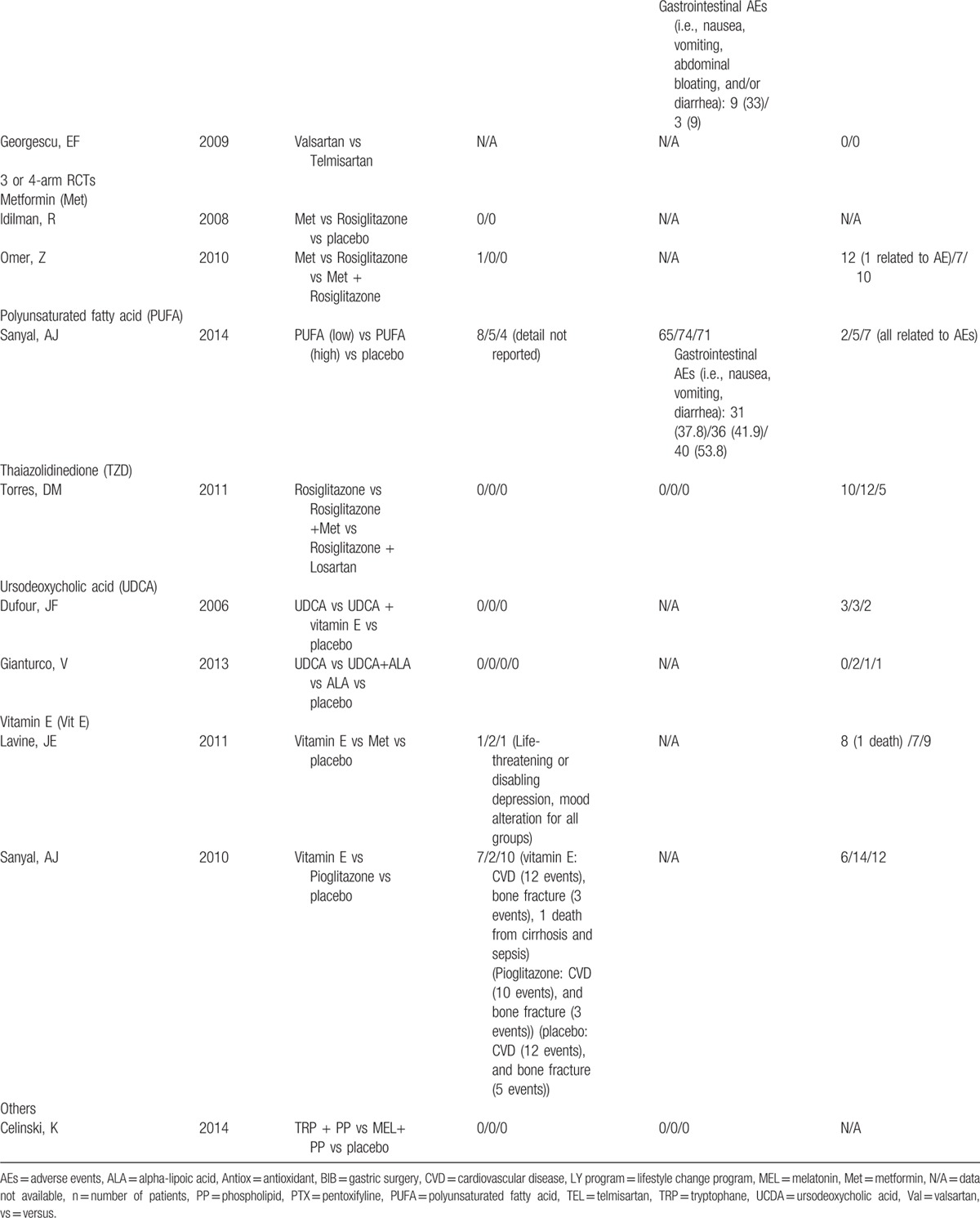
Adverse events and rates of participant dropout of all include studies.

### 3.2 Death outcome

Only 4 studies reported death outcome.[ ^8^ ^41^ ^46^ ^58^] All of them follow up patients more than 1 year. Among 2 studies investigating vitamin E, only 2 cases of death were reported in among vitamin E users (2/142).[ ^41^ ^58^] Cause of death was cirrhosis with sepsis for 1 patient but another was not specified. One study reported death of 2 patients receiving OCA (2/141), 1 died from sepsis with congestive heart failure (CHF), and another one from myocardial infraction (MI), respectively. ^[8]^ In a study of UCDA, 1 patient died from myocardial infarction but it was not specified as a user of UCDA or placebo ^[46]^ (Table 2 ).

### 3.3 Improvement of fibrosis

**Figure 2:**
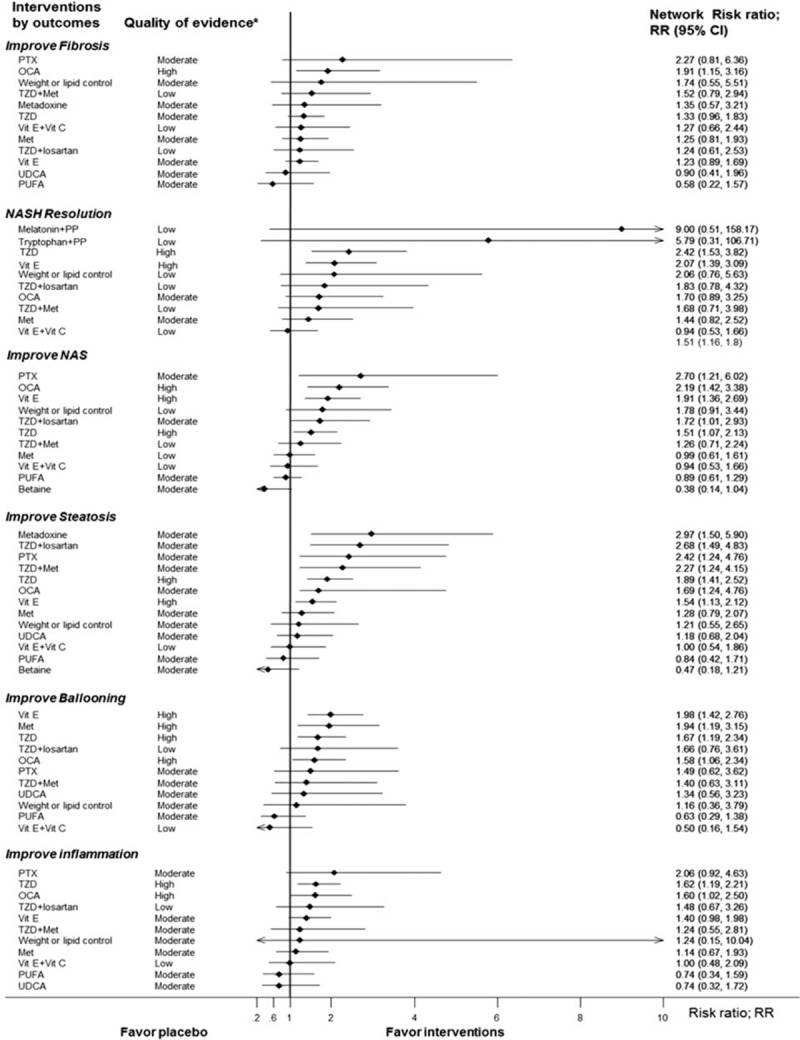
Forest plot summary of network estimates of interventions compared with placebo (cointervention: advise of weight and diet control) on histological outcomes. A, Fibrosis. B, Resolution of NASH (nonalcoholic steatohepatitis). C, NAFLD activity score (NAS). D, Steatosis. E, Ballooning degeneration. F, Lobular inflammation. ^∗^Quality of evidence was graded based on GRADE Working Group: High = we are very confident that the true effect lies close to that of the estimate of the effect, Moderate = we are moderately confident in the effect estimate: the true effect is likely to be close to the estimate of the effect, but there is a possibility that it is substantially different, Low = our confidence in the effect estimate is limited: the true effect may be substantially different from the estimate of the effect, Very low = we have very little confidence in the effect estimate: the true effect is likely to be substantially different from the estimate of effect. NAS = nonalcoholic fatty liver disease (NAFLD) activity score, NASH = nonalcoholic steatohepatitis, PP = phospholipid, PTX = pentoxifyline.

A total of 21 studies[ ^8^ ^29^ ^30^ ^31^ ^33^ ^38^ ^39^ ^40^ ^41^ ^42^ ^46^ ^48^ ^51^ ^55^ ^58^ ^61^ ^65^ ^66^ ^67^ ^69^ ^70^] (n = 1939 patients) reported improvement in fibrosis from 12 interventions (Fig. 1A). Results of direct comparisons showed that only OCA and TZD significantly improved fibrosis relative to placebo, with a pooled RR of 1.91 (1.15, 3.16) and 1.42 (1.01, 1.99), respectively (Appendix Table 3). A network meta-analysis indicated that only OCA remained significant similar effect to the direct one with pooled RR of 1.91(1.15, 3.16) (Fig. 2). PTX, TZD plus Met, weight/lipid control were effective when compared with placebo but these were not significant with a pooled RRs of 2.27 (0.81, 6.36), 1.52 (0.79, 2.94), and 1.74 (0.55, 5.51), respectively. The summary results of network meta-analysis and ranking are reported in Table 3. PTX showed a trend for better than other interventions with the RRs ranging from 1.19 to 3.85, but it was not significant (Table 3).). The ranking of interventions for this outcome can be also found in Appendix Figure 3A.

**Table 3:**
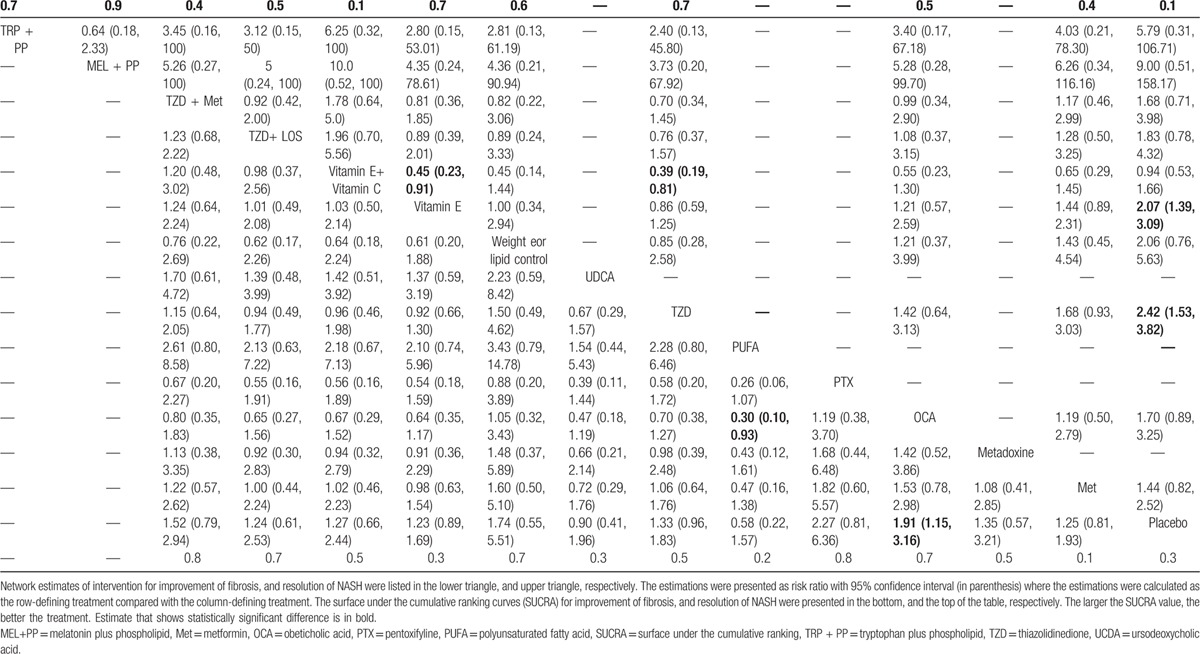
Network meta-analysis estimated effects of intervention for NAFLD therapy on improvement of fibrosis (lower triangle) and resolution of NASH (upper triangle).

### 3.4 Resolution of NASH

Seven studies (n = 1007)[ ^8^ ^32^ ^41^ ^51^ ^54^ ^58^ ^65^] reported resolution of NASH for 11 interventions (Fig. 1B). The direct evidence demonstrated statistically significant higher resolution of NASH for TZD and vitamin E when compared with placebo, with RRs of 2.28 (1.35, 3.87) and 2.07 (1.39, 3.09), respectively (Appendix Table 3). These RRs were not much changed by a network meta-analysis; melatonin/phospholipid and tryptophan/phospholipid additionally showed a trend for efficacy when compared with placebo with a pooled RR of 9.00 (0.51, 158.17), and 5.79 (0.31, 106.71), respectively (Table 3). The ranking of interventions for this outcome can be also found in Appendix Figure 3B.

### 3.5 Improvement of NAFLD activity score (NAS)

Fifteen studies (n = 1590)[ ^8^ ^28^ ^33^ ^39^ ^41^ ^42^ ^48^ ^51^ ^54^ ^55^ ^57^ ^58^ ^65^ ^67^ ^69^] reported improvement of NAS (Fig. 1C). The direct effects of PTX, OCA, TZD, and vitamin E were statistically significant in improvement of NAS when compared with placebo, with RRs of 2.70 (1.21, 6.03), 2.19 (1.42, 3.28), 1.56 (1.08, 2.26), and 2.24 (1.52, 3.31), respectively (Appendix Table 3). A network meta-analysis yielded similar effects but additionally indicated that TZD/losartan showed a significantly higher likelihood of NAS score improvement than placebo with RR of 1.72 (1.01, 2.93) (Fig. 2). The summary results of network meta-analysis and ranking were shown in Appendix (Appendix Table 4 and Appendix Figure 3C).

### 3.6 Improvement of steatosis, ballooning, and lobular inflammation

The summary results of meta-analysis of steatosis, ballooning, and lobular inflammation improvement are reported in Appendix Table 3, while network meta-analysis results are reported in Fig. 2. The direct evidence and network meta-analysis provided similar interventions effects on improvement of steatosis. When compared with placebo, the network meta-analysis RRs for PTX, OCA, TZD, metadoxine, and vitamin E were 2.42 (1.24, 4.76), 1.69 (1.24, 4.76), 1.89 (1.41, 2.52), 2.97 (1.50, 5.90), and 1.54 (1.13, 2.12), respectively. For the improvement of ballooning, both pairwise and network meta-analysis results demonstrated that OCA and vitamin E were significantly better than placebo. The network meta-analysis RRs of those were 1.58 (1.06, 2.34), and 1.98 (1.42, 2.76), respectively (Fig. 2). For improvement of lobular inflammation, both evidences demonstrated that TZD and OCA were significantly better than placebo. The network meta-analysis RRs of those were 1.62 (1.19, 2.21) and 1.60 (1.02, 2.50), respectively (Fig. 2). The ranking of these outcomes can be found in Appendix Figure 3D–F.

### 3.7 Mean change in fibrosis stage, NAS, steatosis, ballooning, and lobular inflammation

All interventions included in network meta-analysis for estimating mean changes of fibrosis stage, NAS, steatosis, ballooning, and lobular inflammation are presented in Appendix Figure 4. Pairwise (direct) meta-analysis of mean changes in fibrosis stage, NAS, steatosis, ballooning, and lobular inflammation are reported in Appendix Table 5, while network meta-analysis results of these outcomes are reported in Appendix Figure 5. Direct comparisons of weighted mean difference of changes in fibrosis grade indicated that PTX, OCA, antioxidant plus UDCA significantly decreased fibrosis grade when compared with placebo, with WMDs of −0.60 (0.95, −0.25), −0.30 (−0.52, −0.08), −0.29 (−0.35, −0.23), respectively (Appendix Table 5). The results remained unchanged in network meta-analysis (Appendix Table 6). Both direct and network meta-analysis results of mean change of NAS, steatosis, ballooning, and lobular inflammation tended to be the same as improvement outcomes (Appendix Table 5, Appendix Figure 5). The ranking efficacy of these outcomes is presented in Appendix Figure 6.

### 3.8 Inconsistency tests

There was no evidence of inconsistency between direct and indirect effects for most outcomes except mean changes in fibrosis stage and NAS (*χ* ^*2*^ = −74.62, *P* value <0.001 for fibrosis change; *χ* ^*2*^ = 89.33, *P* value <0.001 for NAS), respectively (Appendix Table 7). The pooled estimates of these outcomes were then based on an inconsistency model. ^[71]^

### 3.9 Assessment of small-study effects

Small-study effects were assessed using adjusted funnel plots (Appendix Figure 7), indicating small-study effect might be present particularly for fibrosis, NAS, steatosis, ballooning outcomes. Distribution of sample size of all included RCTs was then explored for each outcome. A sensitivity analysis was performed by including only RCTs where their sample sizes exceeded the 25th percentile. Therefore, 10, 5, 6, 9, 8, and 7 trials were included in sensitivity analyses for improvement of fibrosis, resolution of NASH, NAS, steatosis, ballooning, and lobular inflammation, respectively. Results for the primary outcome are described in Appendix Table 8, while secondary outcomes are described in Appendix Table 9. These suggested that most rankings remained the same except the ranking of PTX, weight or lipid control, betaine, tryptophan/phospholipid, and melatonin/phospholipid were omitted.

### 3.10 Quality of evidence

Evidence quality was graded for both network (Fig. 2, Appendix Figure 5) and pairwise (Appendix Table 3, Appendix Table 5) meta-analyses, indicating high quality evidence for OCA in improvement of fibrosis, NAS, ballooning and lobular inflammation, and TZD and vitamin E in resolution of NASH, improvement of NAS, steatosis, ballooning, and lobular inflammation, respectively. Evidence quality for PTX in improvement of NAS and steatosis was moderate (Fig. 2).

### 3.11 Sensitivity and subgroup analyses

Most results from sensitivity and subgroup analyses were comparable with those in main analyses for most interventions (data not shown). The effects of TZD, vitamin E, and PTX on improvement of steatosis disappeared in pooling high quality RCTs (Appendix Table 8). Subgroup analysis showed no significant effects on improvement of any histological outcomes if follow-up time less than 1 year, whereas the effects of PTX on improvement of NAS and steatosis, and vitamin E on lobular inflammation were reversed from the main results (Appendix Table 8).

### 3.12 Adverse events

Adverse events were reported in 34 studies (77%) but only 11 studies reported treatment-related serious/intolerance adverse events. Five of 11 studies reported serious or intolerance adverse events including cardiovascular diseases and peripheral edema related to pioglitazone or rosiglitazone (TZD) more than placebo. Gastrointestinal adverse events including nausea/vomiting, abdominal cramps, bloating, and heartburn were commonly reported in patients who used PTX, PUFA, and betaine than those in patients who used placebo. For other interventions, both serious/intolerance and common adverse events were infrequent and comparable to placebo (Table 2 ).

## 4 Discussion

We conducted a systematic review and network meta-analysis of all published RCTs with biopsy-proven NAFLD to provide a critical summary of evidence of all interventions for NAFLD therapy. Our findings demonstrated that several interventions significantly improved histological outcomes, such as fibrosis and resolution of NASH. Given an increasing trend of NAFLD prevalence globally, our review is timely and clinically relevant for guiding clinical practice of NAFLD management.

OCA was the only intervention that significantly improved fibrosis with a high quality of evidence and suggested other interventions (i.e., PTX, TZD plus metformin, TZD plus losartan) might potentially be effective. TZD and vitamin E resulted in resolution of NASH with high quality of evidence. PTX, OCA, vitamin E, and TZD were effective in improving the NAS score with a moderate quality for PTX and high quality of evidence for the rest.

Our findings were different from those reported in a previous network meta-analysis, which supported efficacy of PTX ^[72]^ not OCA, in improvement of fibrosis. Since the evidence of OCA is supported by a single RCT, while PTX is based on 2 small RCTs with low event rates, the estimated CIs for both treatments were wide. Therefore, there is a need for more RCTs assessing the long-term outcomes and safety of both for NAFLD.

Vitamin E and TZD were supported by high quality of evidence in resolution of NASH. These findings reinforce the current recommendation of American Association for the Study of Liver Diseases (AASLD) guidelines for the use of natural vitamin E (800 IU/day) and pioglitazone in nondiabetic adults with biopsy-proven NASH. ^[1]^ However, concern has been raised about risks of vitamin E therapy. [^73^ ^74^ ^75^ ^76^] Currently, pioglitazone is the only TZD available in clinical practice, because rosiglitazone is not available in Europe and highly restricted in the United States. ^[77]^ The long-term safety of pioglitazone regarding cardiovascular disease (especially chronic heart failure) limits widespread use. ^[78]^ It is important to note that majority of the patients in our included trials is nondiabetic, limiting the applicability of these interventions in diabetic patients.

Our study had a number of advantages over previously published meta-analysis studies of NAFLD.[ ^9^ ^72^ ^79^ ^80^ ^81^] We included both NAFL and NASH patients, assessed both nonpharmacological and pharmacological interventions, and considered only biopsy-proved histological outcomes. Contrastingly, the previous studies only considered NASH patients, assessed only pharmacological interventions, ^[72]^ or considered surrogate outcomes (e.g., liver fatty content by ultrasound, ALT, AST, insulin sensitivity).[ ^9^ ^79^ ^80^ ^81^] The number of RCTs or patients included in our study was larger than the previous published report, ^[72]^ that is, 44 and 3802 versus 9 and 964, respectively. A unique feature of our study is the inclusion of reports in patients with NASH and NAFLD, which provides a more global assessment of therapeutic interventions in this disease state.[ ^4^ ^82^]

Limitations of our study are the heterogeneity from inclusion of various interventions and patient characteristics and the fact that a large number (60%) of the included studies were rated as unclear/high ROB, although sensitivity and subgroup analyses showed similar results to the main findings. In addition, more relevant long-term outcomes (e.g., cirrhosis, death, safety) could be not assessed because none of included studies reported. Furthermore, number of included studies and subjects were very small and thus yielded imprecise estimation of some treatment effects. These results are thus needed to update when there are more RCTs available.

In conclusion, we observed that of the interventions studies thus far, OCA was effective for improving fibrosis and NAS score, while TZD and vitamin E were effective for resolution of NASH and NAS score. Large comparative RCTs and cost-effectiveness analyses are warranted to investigate the effects of interventions on histological and clinical outcomes.

## Supplementary Material

Supplemental Digital Content
