# Supplementary material for: Comparative efficacy of interventions on nonalcoholic fatty liver disease (NAFLD): A PRISMA-compliant systematic review and network meta-analysis
Source: Medicine (Baltimore). 2016 Aug 12;95(32):e4529. doi: 10.1097/MD.0000000000004529 (PMC4985329; doi:10.1097/MD.0000000000004529)
Supplement: Supplemental Digital Content [file medi-95-e4529-s001.pdf]

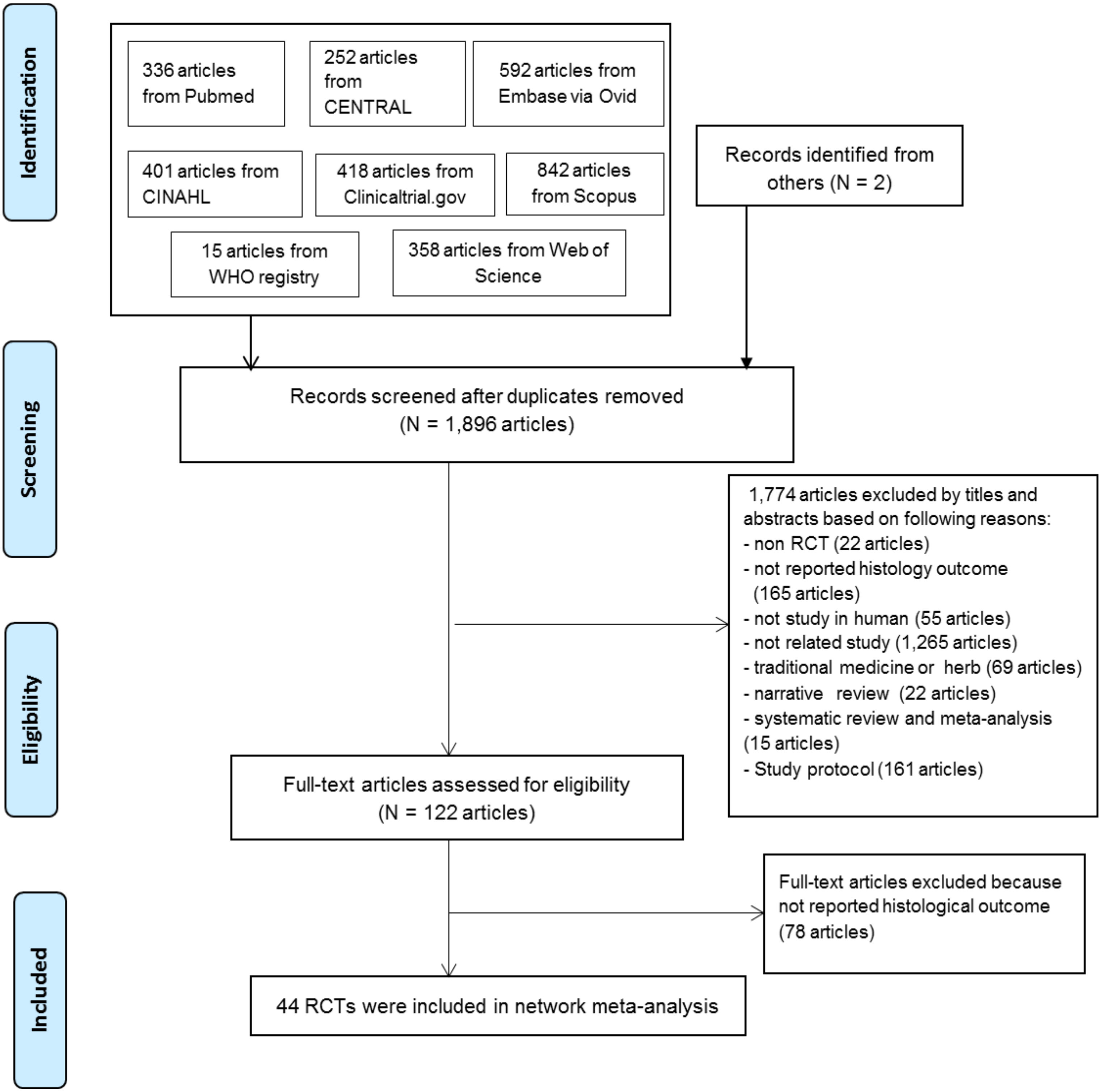

Appendix Figure 1. A flow of study selection

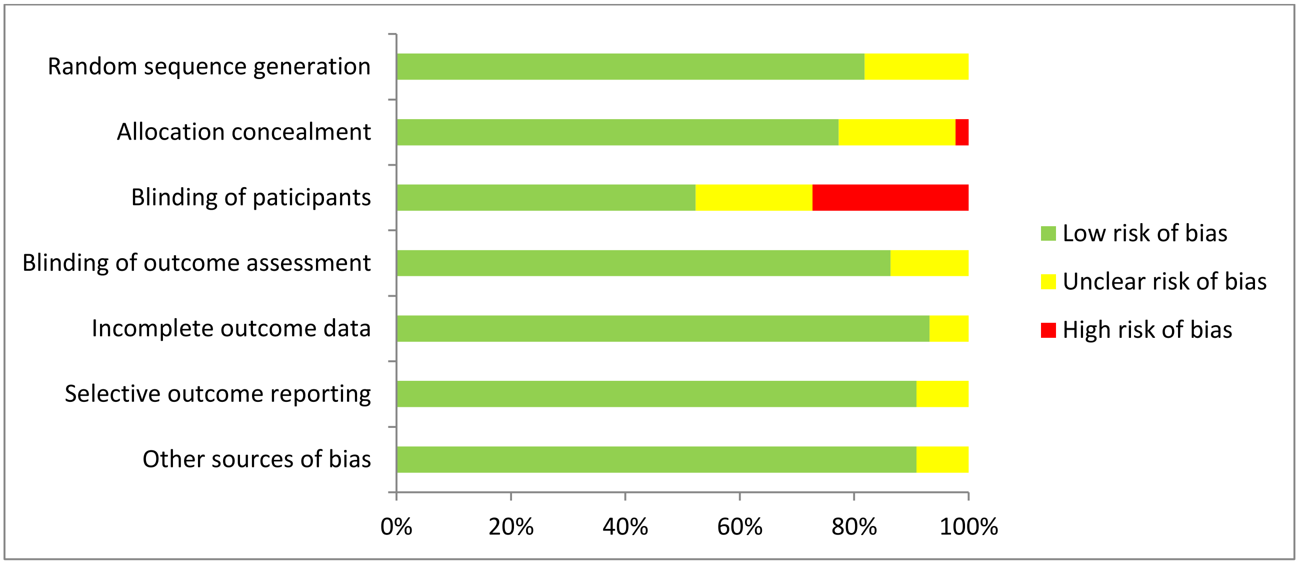

A.

B.

Adequate sequence generation?	Allocation concealment?	Blinding patient?	Blinding assessor?	Incomplete outcome data?	Selective outcome reporting?	Other sources of bias?	Author, year
?	+	?	+	+	+	+	Abdelmalek MF, 2009
+	+	?	+	+	+	+	Aithal GP, 2008
+	+	?	+	+	+	+	Belfort R, 2006
?	?	-	?	?	?	?	Celinski K, 2014
+	+	+	+	+	+	+	Dasarathy S, 2015
+	+	+	+	+	+	+	Dufour JF, 2006
+	+	?	+	+	+	+	Georgescu EF, 2009
+	+	+	+	+	+	+	Gianturco V, 2013
+	+	-	+	+	+	+	Gomez EV, 2009
+	+	+	?	+	+	+	Lee YM,2012
+	+	-	?	+	+	+	Han Y, 2013
+	+	+	+	+	+	+	Harrison SA, 2003
+	+	+	+	+	+	+	Haukeland JW, 2009
**+**	**?**	**-**	+	+	+	+	**Idilman R, 2008**
+	+	+	+	+	+	+	Lavine JE, 2011
+	+	+	+	+	+	+	Le TA, 2012
?	+	+	+	+	+	+	Leuschner UFH, 2010
?	?	-	?	?	?	?	Li YH, 2015
+	+	+	+	+	+	+	Lindor KD, 2004
+	+	+	+	+	+	?	Loguercio C, 2012
+	+	+	+	+	?	+	Malaguarnera M, 2010
?	?	?	+	+	?	?	Nelson A, 2009
+	+	+	+	+	+	+	Tetri BAN, 2015
+	+	-	+	+	+	+	Nobili V, 2008
?	?	-	+	+	+	+	Omer Z, 2010
+	+	-	+	+	+	+	Promrat K, 2010

Adequate sequence generation?	Allocation concealment?	Blinding patient?	Blinding assessor?	Incomplete outcome data?	Selective outcome reporting?	Other sources of bias?	Author, year
+	+	?	+	+	+	+	Ratziu V, 2008
?	?	?	+	+	+	+	Rinella ME, 2009
+	?	+	+	+	+	+	Sanyal AJ, 2004
+	+	+	+	+	+	+	Sanyal AJ, 2010
+	+	+	+	+	+	+	Sharma BC, 2012
+	+	-	+	+	+	+	Shields WW, 2009
+	+	-	+	+	+	+	Takeshita Y, 2014
+	+	-	+	+	+	+	Torres DM, 2011
+	-	-	?	+	+	+	Uygun A, 2004
+	+	+	+	+	+	+	Van Wagner LB, 2011
+	+	+	+	+	+	+	Zein CO, 2012
+	+	+	+	+	+	+	Zelber-sagi S, 2006
?	?	?	+	+	+	+	Sturm N, 2009
+	+	+	+	+	+	+	Shenoy KT, 2014
+	+	+	+	+	+	+	Argo, CK.
+	+	+	+	+	+	+	Nogueira MA, 2015
+	+	+	+	?	+	+	Loomba R, 2015
+	?	?	?	+	+	+	Sanyal AJ, 2014

Appendix Figure 2. Summary (A) and individual (B) risk of bias (ROB) of all included studies.

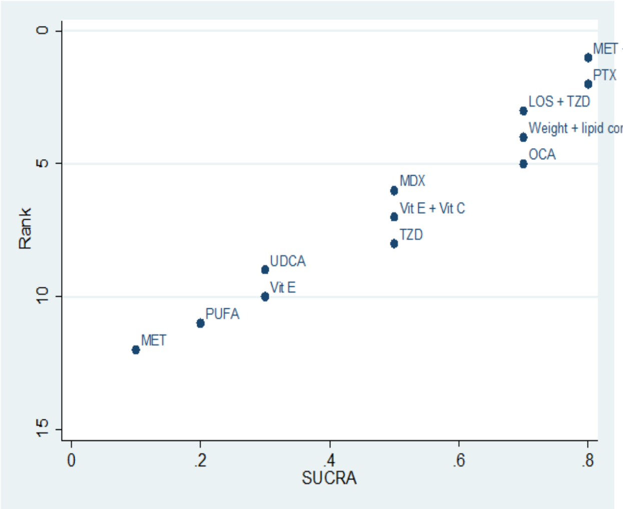

A. Fibrosis

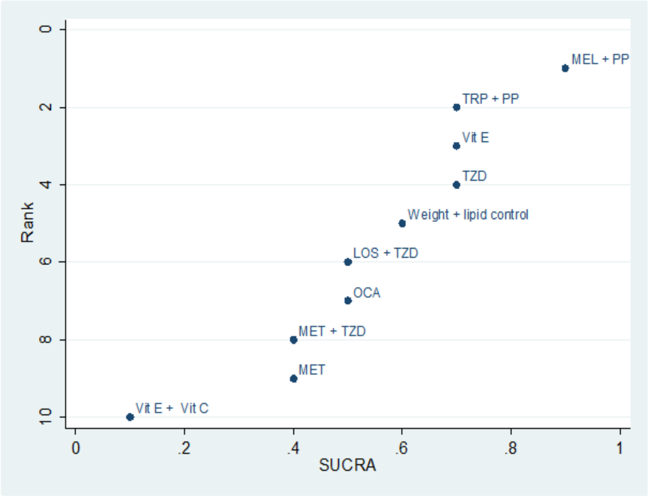

B. Resolution of NASH

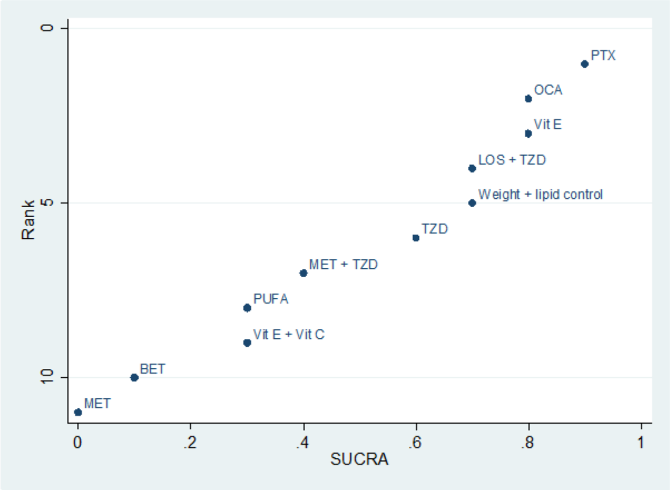

C. NAFLD Activity score (NAS)

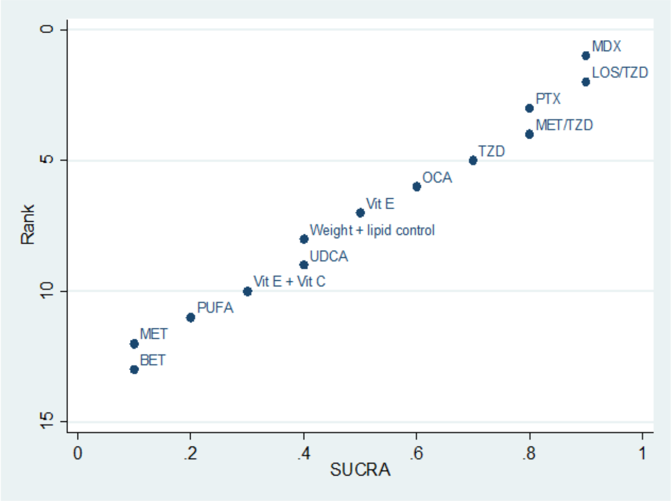

D. Steatosis

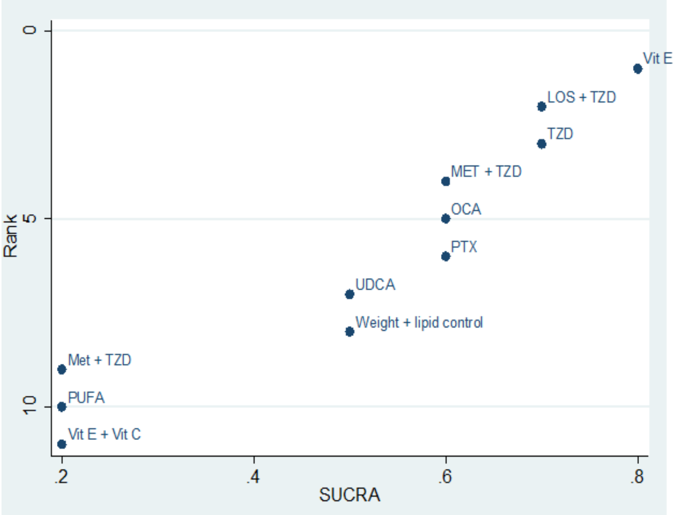

E. Ballooning

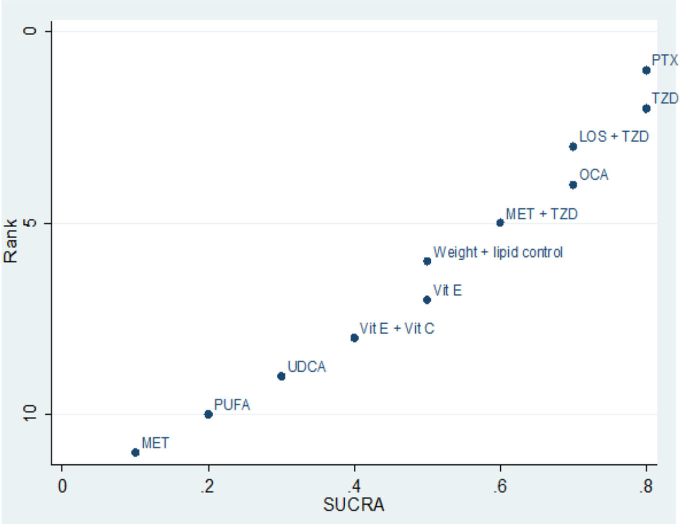

F. Lobular inflammation

Appendix Figure 3. Rank probability of interventions on improvement of histological outcomes (Rank vs. SUCRA); A. Fibrosis, B. Resolution of NASH (Non-alcoholic steatohepatitis); C. NAFLD Activity score (NAS), D. Steatosis, E. Ballooning degeneration, and F. Lobular inflammation

A. Fibrosis

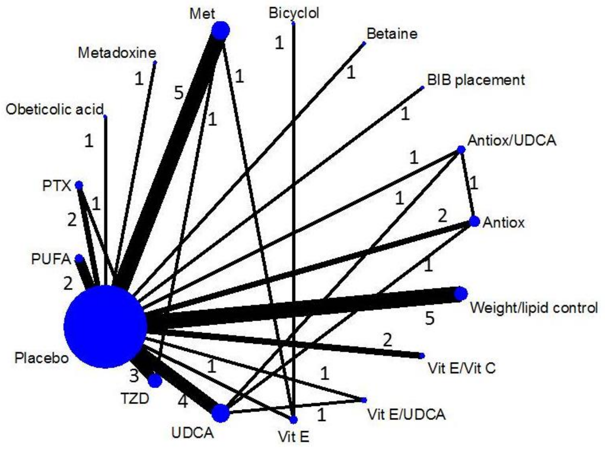

B. NAFLD Activity score (NAS)

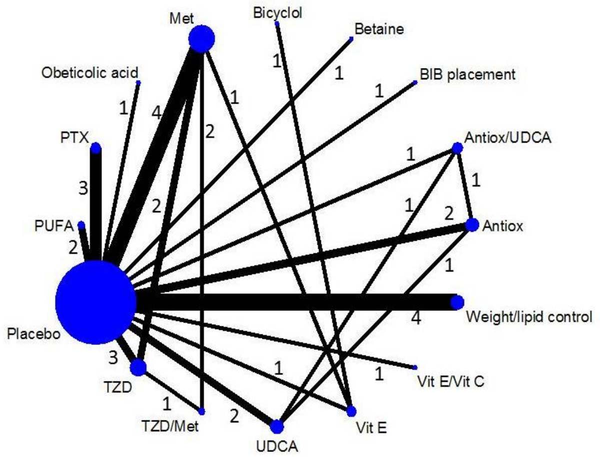

C. Steatosis

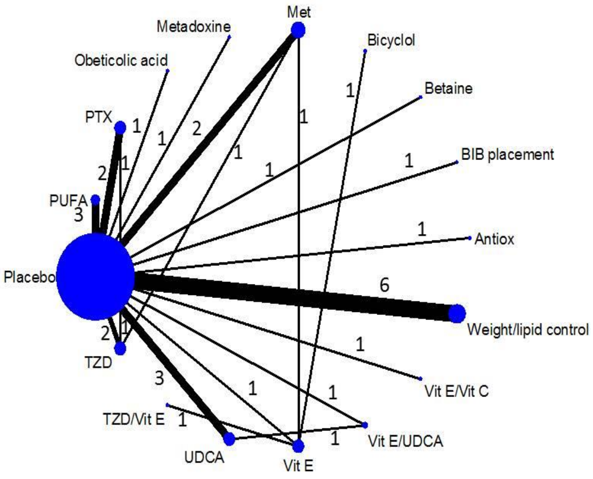

D. Ballooning

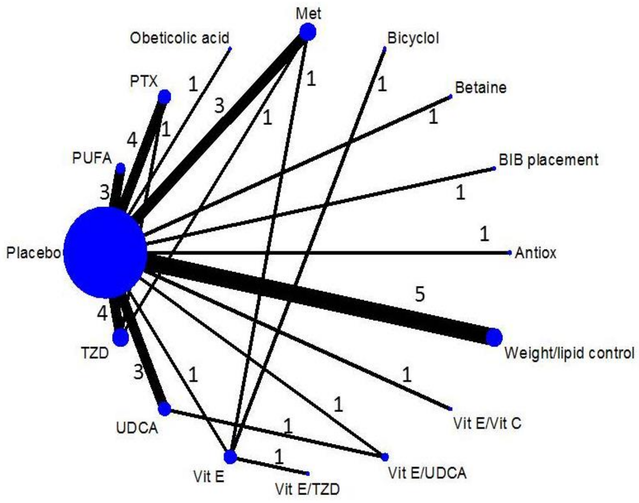

E. Lobular inflammation

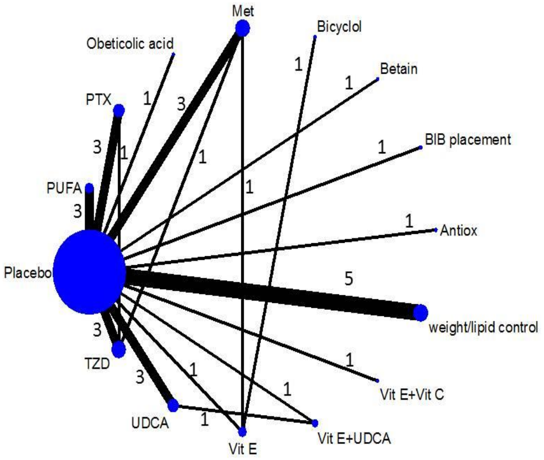

Appendix Figure 4. Network map for mean changes of histological outcomes; A. Fibrosis, B. NAFLD Activity score (NAS), C. Steatosis, D. Ballooning degeneration, and E. Inflammation.

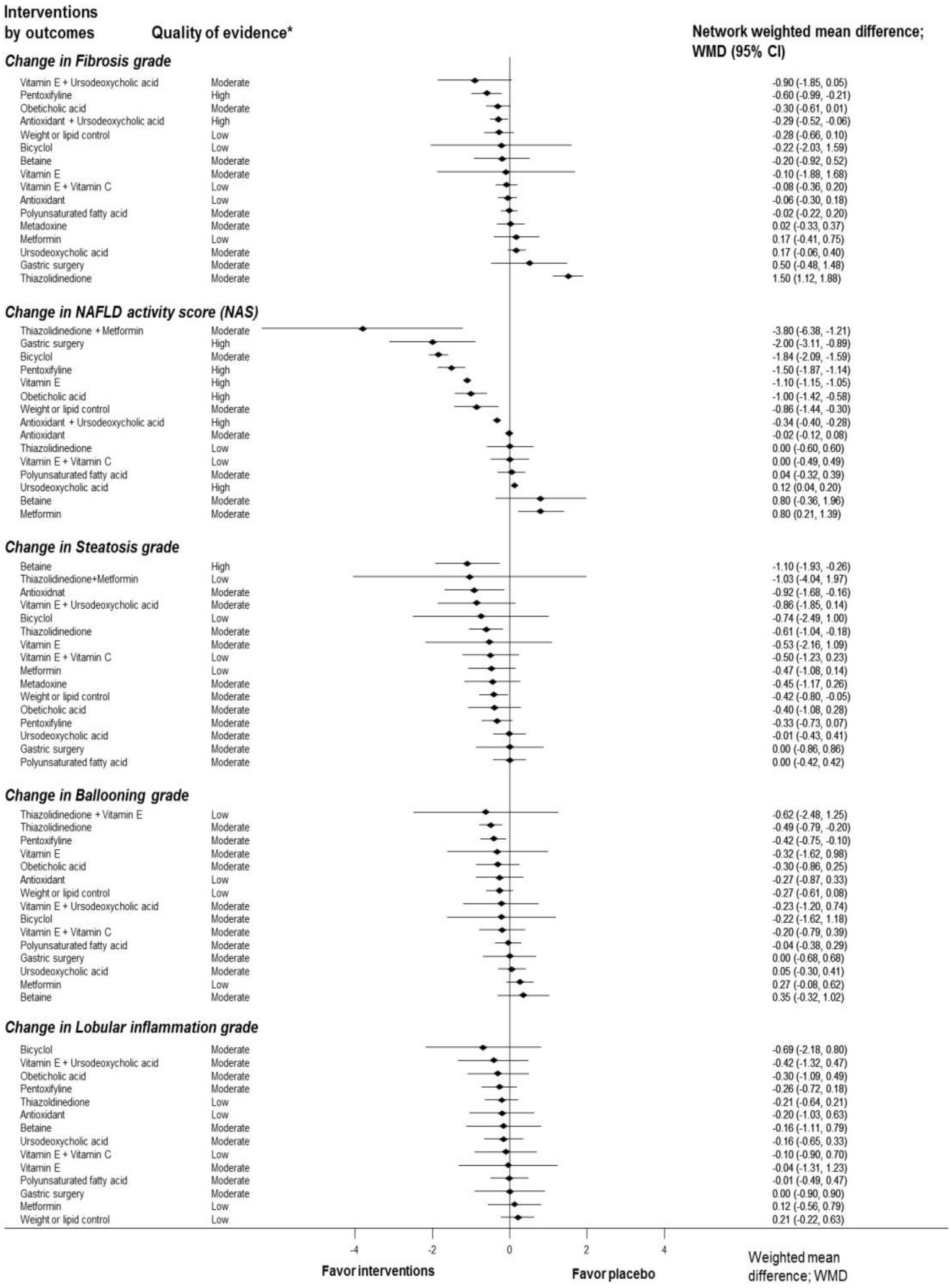

Appendix Figure 5. Summary of network estimates of interventions compared with placebo (co-intervention: advise of weight and diet control) on weighted mean difference (WMD) of histological outcomes; Please note that in the forest plot, ‘experimental’ refers to first treatment group, whereas ‘control’ refers to the second treatment group.

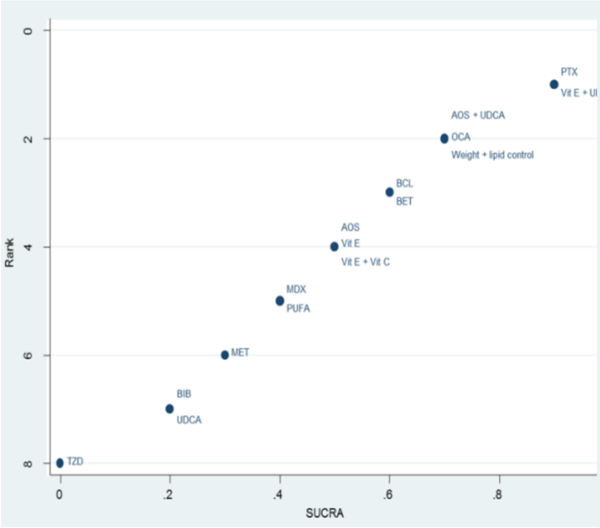

A. Fibrosis

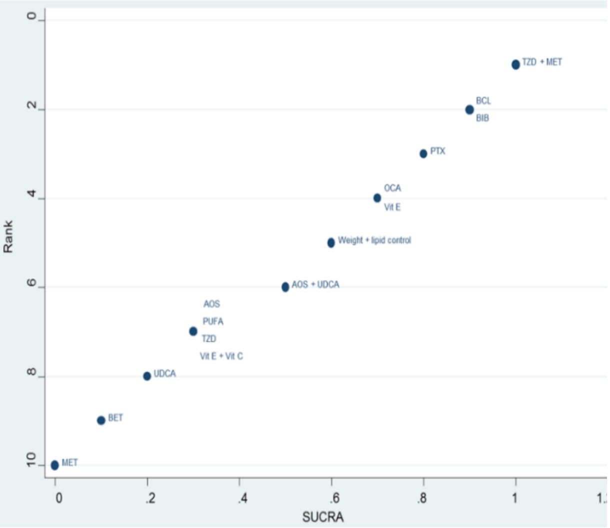

B. NAFLD Activity score (NAS)

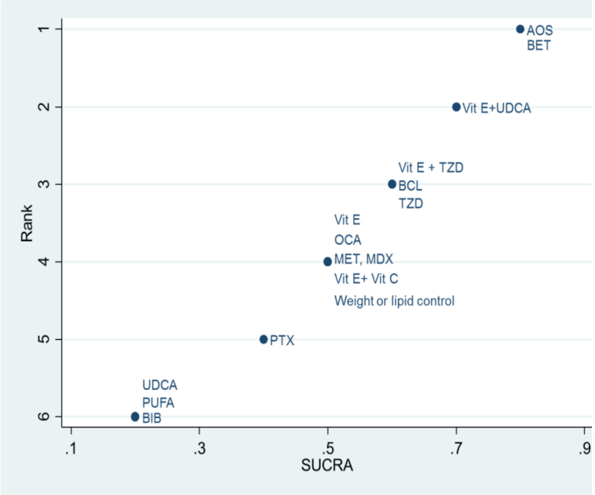

C. Steatosis

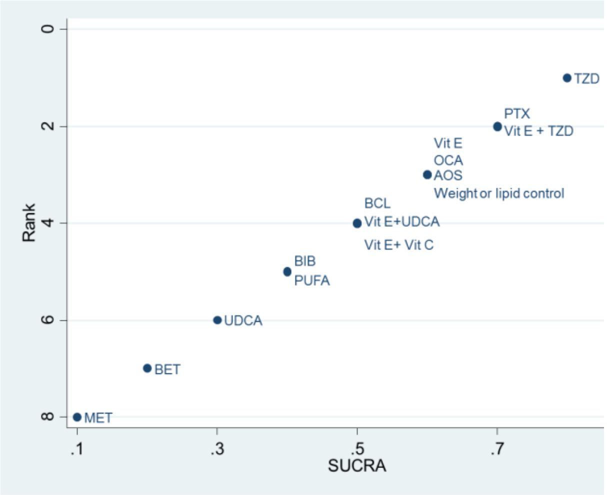

D. Ballooning

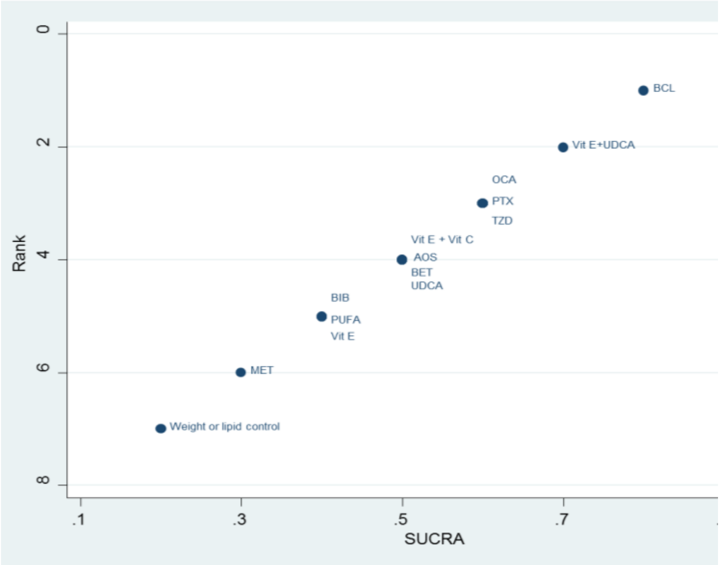

E. Lobular inflammation

Appendix Figure 6. Rank probability of interventions on mean change of histological outcomes (Rank vs. SUCRA); A. Fibrosis, B. NAFLD Activity score (NAS), C. Steatosis, D. Ballooning degeneration, and E. Lobular inflammation.

A. Fibrosis (all studies)

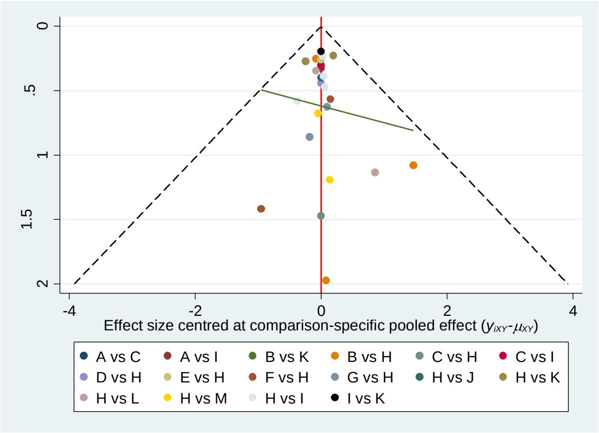

A. Fibrosis (after small studies omitted)

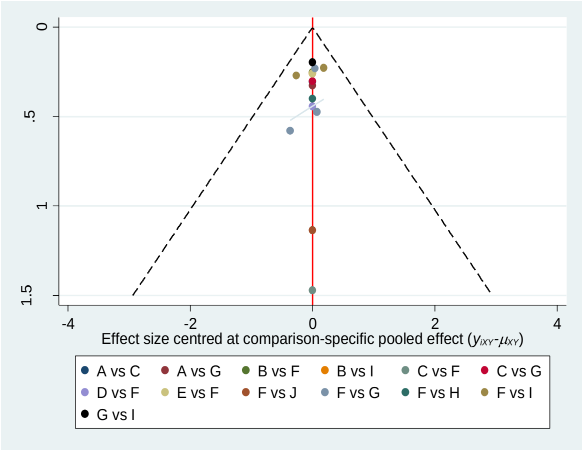

B. Resolution of NASH (all studies)

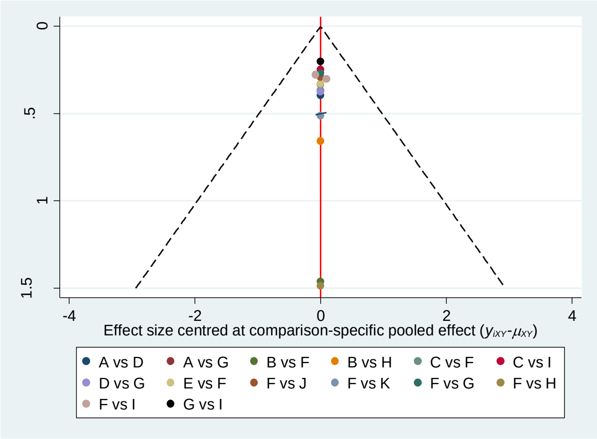

B. Resolution of NASH (after small studies omitted)

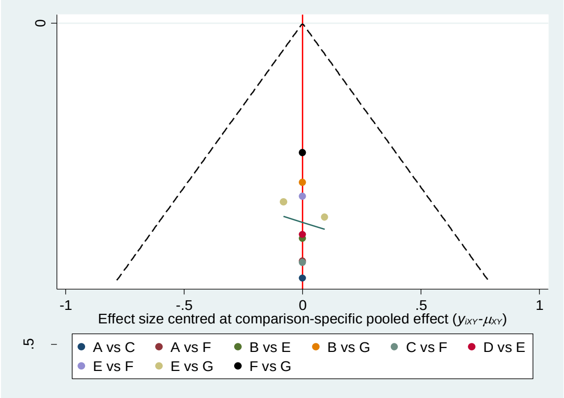

C. NAS (all studies)

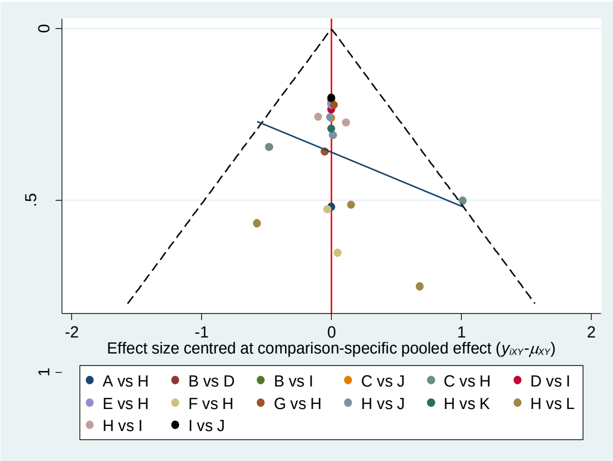

C. NAS (after small studies omitted)

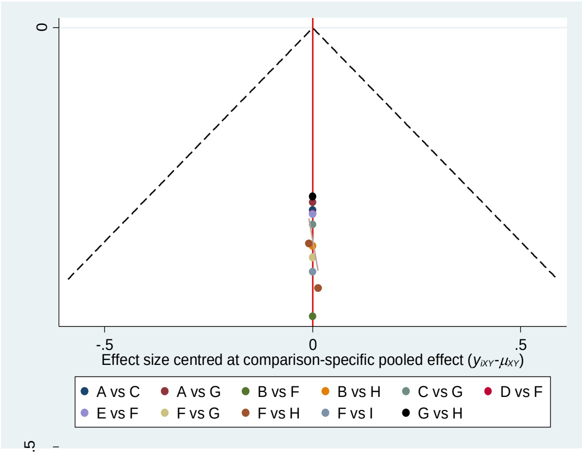

D. Steatosis (all studies)

D. Steatosis (after small studies omitted)

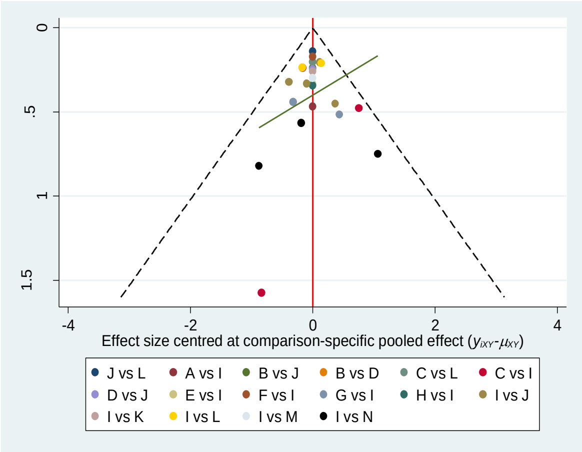

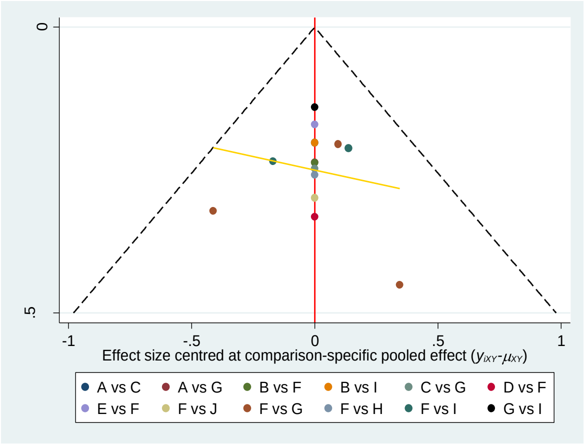

E. Ballooning (all studies)

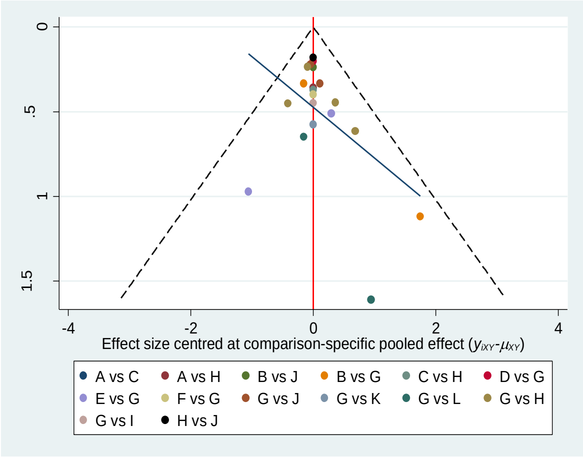

E. Ballooning (after small studies omitted)

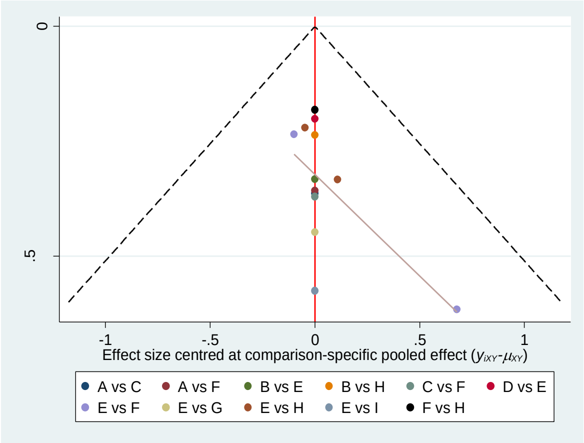

F. Lobular inflammation (all studies)

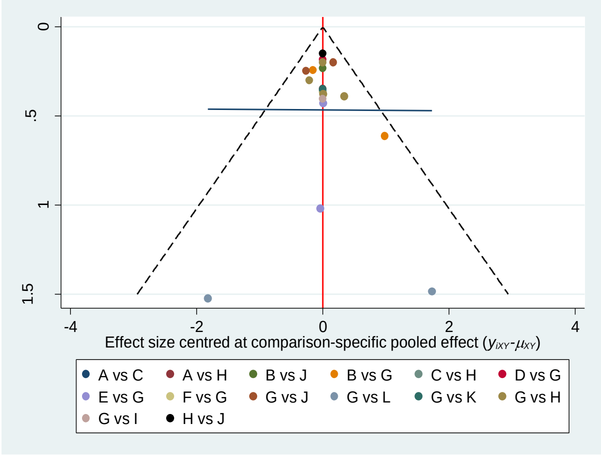

G. Lobular inflammation (after small studies omitted)

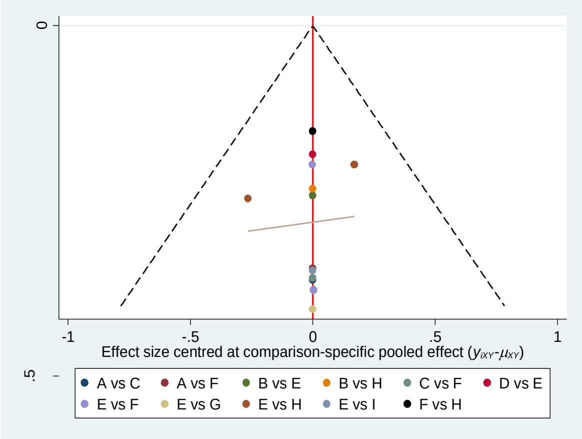

Appendix Figure 7. Comparison adjusted funnel plots of improvement outcomes in the network of interventions used for treatment NAFLD of all studies and after small studies was omitted; A. Fibrosis, B. Resolution of NASH, C. NAS, D. Steatosis, E. Ballooning, and F. Lobular inflammation

Appendix Table 1. Example of searching

Databases		Key word and strategies
**PubMed**		Non-alcoholic fatty liver disease Filters: Clinical Trials
**Cochrane Central Register of Controlled Trials (CENTRAL)**	#1	Non-alcoholic fatty liver disease.mp. [mp=title, original title, abstract, mesh headings, heading words, keyword]
	#2	Fatty liver.mp. [mp=title, original title, abstract, mesh headings, heading words, keyword]
	#3	Clinical trial.mp. [mp=title, original title, abstract, mesh headings, heading words, keyword]
	#4	#1 or #2
	#5	#3 and #4
**EMBASE**	1	Non-alcoholic fatty liver disease.mp. [mp=title, abstract, heading word, drug trade name, original title, device manufacturer, drug manufacturer, device trade name, keyword]
	2	Randomized controlled trial.mp. [mp=title, abstract, heading word, drug trade name, original title, device manufacturer, drug manufacturer, device trade name, keyword]
	3	RCT.mp. [mp=title, abstract, heading word, drug trade name, original title, device manufacturer, drug manufacturer, device trade name, keyword]
	4	Clinical trial.mp. [mp=title, abstract, heading word, drug trade name, original title, device manufacturer, drug manufacturer, device trade name, keyword]
	5	Clinical trials.mp. [mp=title, abstract, heading word, drug trade name, original title, device manufacturer, drug manufacturer, device trade name, keyword]
	6	2 or 3 or 4 or 5
	7	1 and 6

Appendix Table 2. GRADE Working Group grades of evidence*

Grading	Meaning
High quality	We are very confident that the true effect lies close to that of the estimate of the effect
Moderate quality	We are moderately confident in the effect estimate: The true effect is likely to be close to the estimate of the effect, but there is a possibility that it is substantially different
Low quality	Our confidence in the effect estimate is limited: The true effect may be substantially different from the estimate of the effect
Very low quality	We have very little confidence in the effect estimate: The true effect is likely to be substantially different from the estimate of effect

*The quality of evidence based on grading system can be rated down based on risk of bias (i.e., low risk, unclear, high risk), indirectness (i.e., measurement outcome such as death (direct outcome) or ALT level (indirect outcome)), imprecision (i.e., wide range of 95%CI), inconsistency (or heterogeneity) and/or publication bias, to levels of moderate, low and very low quality.

Appendix Table 3. Pairwise (direct) estimates effects of different interventions compared with placebo (co-intervention: advise of weight and diet control) on improvement of histological outcome (outcomes reported as risk ratio (RR) along with 95%CI in parenthesis) with quality of evidence

Intervention	N (study)	Fibrosis	GRADE	N (study)	Resolution of NASH	GRADE	N (study)	NAS	GRADE	n (study)	Inflammation	GRADE	n (study)	Ballooning	GRADE	n (study)	Steatosis	GRADE
**Placebo control**																		
PTX	85 (2)	2.27 (0.81, 6.36)	⊕⊕⊕◯ MODERATE [1]	N/A	N/A	N/A	85 (2)	**2.70 (1.21, 6.03)**	⊕⊕⊕◯ MODERATE [1]	83 (2)	2.06 (0.95, 4.48)	⊕⊕⊕◯ MODERATE [1]	85 (2)	1.73 (0.48, 6.24)	⊕⊕⊕◯ MODERATE [1]	85 (2)	**2.40 (1.15, 5.03)**	⊕⊕⊕◯ MODERATE [1]
Weigh or lipid control	152 (3)	2.00 (0.68, 5.85)	⊕⊕⊕◯ MODERATE [1]	31 (1)	2.06 (0.76, 5.63)	⊕⊕◯◯ LOW [1,2]	131 (3)	1.77 (0.91, 3.44)	⊕⊕◯◯ LOW [1,2]	100 (2)	1.20 (0.04, 39.31)	⊕⊕◯◯ LOW [1,3]	100 (2)	1.17 (0.36, 3.79)	⊕⊕⊕◯ MODERATE [1]	152 (2)	1.22 (0.44, 3.37)	⊕⊕⊕◯ MODERATE [1]
**Obeticholic acid**	283 (1)	**1.91 (1.15, 3.16)**	⊕⊕⊕⊕ HIGH	283 (1)	1.70 (0.89, 3.25)	⊕⊕⊕◯ MODERATE[1]	183 (1)	**2.19 (1.42, 3.28)**	⊕⊕⊕⊕ HIGH	283 (1)	**1.60 (1.12, 2.29)**	⊕⊕⊕⊕ HIGH	283 (1)	**1.58 (1.06, 2.34)**	⊕⊕⊕⊕ HIGH	283 (1)	**1.69 (1.21, 2.36)**	⊕⊕⊕⊕ HIGH
**TZD**	354 (4)	**1.42 (1.01, 1.99)**	⊕⊕⊕⊕ HIGH	163 (1)	**2.28 (1.35, 3.87)**	⊕⊕⊕⊕ HIGH	226 (2)	**1.56 (1.08, 2.26)**	⊕⊕⊕⊕ HIGH	354 (4)	**1.74 (1.32, 2.29)**	⊕⊕⊕⊕ HIGH	354 (1)	1.53 (0.97, 2.43)	⊕⊕⊕◯ MODERATE [1]	354 (4)	**2.01 (1.52, 2.67)**	⊕⊕⊕⊕ HIGH
Metadoxine	134 (1)	1.35 (0.57, 3.21)	⊕⊕⊕◯ MODERATE [1]	N/A	N/A	N/A	N/A	N/A	N/A	N/A	N/A	N/A	N/A	N/A	N/A	134 (1)	**2.97 (1.55, 5.70)**	⊕⊕⊕⊕ HIGH
Vit E	283 (2)	1.21 (0.79, 1.88)	⊕⊕⊕◯ MODERATE [1]	267 (2)	**2.07 (1.39, 3.09)**	⊕⊕⊕⊕ HIGH	**283 (2)**	**2.24 (1.52, 3.31)**	⊕⊕⊕⊕ HIGH	167 (1)	**1.70 (1.15, 2.51)**	⊕⊕⊕⊕ HIGH	283 (2)	**1.97 (1.38, 2.83)**	⊕⊕⊕⊕ HIGH	283 (2)	**1.68 (1.24, 2.29)**	⊕⊕⊕⊕ HIGH
UDCA	166 (1)	0.90 (0.41, 1.96)	⊕⊕⊕◯ MODERATE [1]	N/A	N/A	N/A	N/A	N/A	N/A	166 (1)	0.74 (0.34, 1.65)	⊕⊕⊕◯ MODERATE[1]	166 (1)	1.34 (0.56, 3.23)	⊕⊕⊕◯ MODERATE [1]	166 (1)	1.18 (0.71, 1.96)	⊕⊕⊕◯ MODERATE [1]
Met	197 (3)	0.76 (0.19, 3.07)	⊕⊕◯◯ LOW [1,2]	97 (1)	1.55 (0.80, 2.98)	⊕⊕⊕◯ MODERATE[1]	163 (2)	0.73 (0.17, 3.16)	⊕⊕◯◯ LOW [1,2]	163 (2)	0.76 (0.25, 2.27)	⊕⊕◯◯ LOW [1,3]	163 (2)	1.16 (0.19, 6.94)	⊕⊕◯◯ LOW [1,3]	182 (3)	1.05 (0.51, 2.16)	⊕⊕⊕◯ MODERATE[1]
PUFA	78 (2)	0.58 (0.22, 1.57)	⊕⊕⊕◯ MODERATE [1]	N/A	N/A	N/A	280 (2)	0.89 (0.61, 1.29)	⊕⊕⊕◯ MODERATE[1]	37 (1)	0.74 (0.36, 1.52)	⊕⊕⊕◯ MODERATE[1]	37 (1)	0.63 (0.29, 1.38)	⊕⊕⊕◯ MODERATE [1]	37 (1)	0.84 (0.43, 1.65)	⊕⊕⊕◯ MODERATE [1]
Betaine	N/A	N/A	N/A	N/A	N/A	N/A	55 (1)	0.38 (0.14, 1.04)	⊕⊕⊕◯ MODERATE[1]	N/A	N/A	N/A	N/A	N/A	N/A	55 (1)	0.47 (0.19, 1.18)	⊕⊕⊕◯ MODERATE [1]
TZD +Met	74 (1)	4.68 (0.26, 83.63)	⊕⊕◯◯ LOW [1,2]	N/A	N/A	N/A	N/A	N/A	N/A	N/A	N/A	N/A	N/A	N/A	N/A	N/A	N/A	N/A
Vit E + Vit C	139 (2)	1.27 (0.66, 2.44)	⊕⊕◯◯ LOW [1,2]	90 (1)	0.94 (0.53, 1.66)	⊕⊕◯◯ LOW [1,2]	90 (1)	0.94 (0.53, 1.66)	⊕⊕◯◯ LOW [1,2]	90 (1)	1.00 (0.50, 1.98)	⊕⊕◯◯ LOW [1,2]	90 (1)	0.50 (0.16, 1.54)	⊕⊕◯◯ LOW [1,2]	90 (1)	1.00 (0.56, 1.79)	⊕⊕◯◯ LOW [1,2]
MEL + PP	N/A	N/A	N/A	46 (1)	9.00 (0.51, 158.17)	⊕⊕◯◯ LOW [1] N/A	N/A	N/A	N/A	N/A	N/A	N/A	N/A	N/A	N/A	N/A	N/A	N/A
TRP +PP	N/A	N/A	N/A	51 (1)	5.79 (0.31, 106.71)	⊕⊕◯◯ LOW [1] N/A	N/A	N/A	N/A	N/A	N/A	N/A	N/A	N/A	N/A	N/A	N/A	N/A
**Active controls (comparators)**																		

Intervention	N (study)	Fibrosis	GRADE	N (study)	Resolution of NASH	GRADE	N (study)	NAS	GRADE	n (study)	Inflammation	GRADE	n (study)	Ballooning	GRADE	n (study)	Steatosis	GRADE
Vit E (vs. TZD)	164 (1)	1.01 (0.69, 1.49)	⊕⊕⊕◯ MODERATE[1]	164 (1)	0.84 (0.56, 1.23)	⊕⊕⊕◯ MODERATE[1]	164 (1)	1.27 (0.86, 2.34)	⊕⊕⊕◯ MODERATE[1]	164 (1)	0.97 (0.72, 1.32)	⊕⊕⊕◯ MODERATE[1]	164 (1)	1.23 (0.86, 1.75)	⊕⊕⊕◯ MODERATE[1]	164 (1)	0.85 (0.65, 1.12)	⊕⊕⊕◯ MODERATE[1]
Vit E (vs. Met)	115 (1)	0.80 (0.49, 1.33)	⊕⊕⊕◯ MODERATE[1]	97 (1)	1.47 (0.90, 2.38)	⊕⊕⊕◯ MODERATE[1]	115 (1)	1.54 (0.92, 2.56)	⊕⊕⊕◯ MODERATE[1]	115 (1)	0.94 (0.60, 1.49)	⊕⊕⊕◯ MODERATE[1]	115 (1)	0.98 (0.62, 1.56)	⊕⊕⊕◯ MODERATE[1]	115 (1)	1.02 (0.68, 1.52)	⊕⊕⊕◯ MODERATE[1]
TZD + LOS (vs. TZD)	86 (1)	0.91 (0.48, 1.73)	⊕⊕◯◯ LOW [1 2]	86 (1)	0.76 (0.37, 1.57)	⊕⊕◯◯ LOW [1 2]	86 (1)	1.14 (0.76, 1.71)	⊕⊕◯◯ LOW [1 2]	86 (1)	0.91 (0.46, 1.80)	⊕⊕◯◯ LOW [1 2]	86 (1)	0.99 (0.49, 2.00)	⊕⊕⊕◯ MODERATE[2]	86 (1)	1.42 (0.90, 2.26)	⊕⊕◯◯ LOW [1 2]
TZD + Met (vs. TZD)	90 (1)	1.09 (0.61, 1.98)	⊕⊕◯◯ LOW [1 2]	90 (1)	0.70 (0.34, 1.45)	⊕⊕◯◯ LOW [1 2]	90 (1)	0.84 (0.53, 1.33)	⊕⊕◯◯ LOW [1 2]	90 (1)	0.77 (0.38, 1.55)	⊕⊕⊕◯ MODERATE [1]	90 (1)	0.84 (0.41, 1.73)	⊕⊕⊕◯ MODERATE [1]	90 (1)	1.20 (0.74, 1.95)	⊕⊕◯◯ LOW [1 2]
TZD + Met (vs. TZD + LOS)	94 (1)	1.20 (0.66, 1.98)	⊕⊕◯◯ LOW [1 2]	94 (1)	0.92 (0.42, 2.00)	⊕⊕◯◯ LOW [1 2]	94 (1)	0.73 (0.48, 1.13)	⊕⊕◯◯ LOW [1 2]	94 (1)	0.84 (0.41, 1.71)	⊕⊕◯◯ LOW [1 2]	94 (1)	0.84 (0.41, 1.71)	⊕⊕◯◯ LOW [1 2]	94 (1)	0.84 (0.57, 1.26))	⊕⊕◯◯ LOW [1 2]

Note: CI = Confidence interval, RR = Risk ratios, GRADE = Levels of quality of evidence using GRADE system approach, Reason for grading; 1. Wide range of 95% CI, 2. At least 1 study has high risk of bias, 3. There is evidence of inconsistency.

Appendix Table 4. League table presenting network meta-analysis estimates (lower triangle) and direct estimates (upper triangle) for improvement of NAS of interventions for NAFLD

Vitamin E + Vitamin C	-	-	-	-	-	-	-	-	-	-	0.94 (0.53, 1.66)
0.74 (0.33,1.67)	TZD + Met	0.73 (0.48, 1.13)	-	-	0.84 (0.53, 1.33)	-	-	-	-	-	-
0.55 (0.25,1.19)	0.73 (0.48,1.13)	TZD + LOS	-	-	1.14 (0.76, 1.71)	-	-	-	-	-	-
0.53 (0.22,1.27)	0.71 (0.30,1.71)	0.97 (0.41,2.27)	Weight or lipid control	-	-	-	-	-	-	-	1.77 (0.91, 3.44)
**0.49 (0.25,0.95)**	0.66 (0.37,1.17)	0.90 (0.53,1.53)	0.93 (0.44,1.95)	Vitamin E	1.27 (0.86, 2.34)	-	-	-	1.54 (0.92, 2.56)	-	**2.24 (1.52, 3.31)**
0.62 (0.32,1.21)	0.84 (0.53,1.33)	1.14 (0.76,1.71)	0.93 (0.44,1.95)	1.27 (0.90,1.80)	TZD	-	-	-	-	-	**1.56 (1.08, 2.26)**
1.05 (0.53,2.08)	1.42 (0.72,2.81)	**1.93 (1.01,3.69)**	1.18 (0.56,2.48)	**2.15 (1.30,3.55)**	**1.69 (1.02,2.81)**	PUFA	-	-	-	-	-
**0.35 (0.13,0.93)**	0.47 (0.17,1.25)	0.64 (0.24,1.67)	1.99 (0.93,4.26)	0.71 (0.30,1.69)	0.56 (0.23,1.34)	0.33 (0.14,0.80)	PTX	-	-	-	**2.70 (1.21, 6.03)**
**0.43 (0.21,0.88)**	0.58 (0.28,1.19)	0.78 (0.39,1.56)	0.66 (0.23,1.86)	0.87 (0.50,1.52)	0.69 (0.40,1.20)	0.41 (0.23,0.72)	1.23 (0.50,3.07)	OCA	-	-	**2.19 (1.42, 3.28)**
0.95 (0.45,2.00)	1.28 (0.63,2.58)	1.74 (0.89,3.40)	0.81 (0.37,1.79)	1.93 (1.22,3.06)	1.52 (0.89,2.60)	0.90 (0.49,1.66)	**2.73 (1.07,6.97)**	**2.21 (1.15,4.25)**	Met	-	0.73 (0.17, 3.16)
2.49 (0.78,7.96)	**3.35 (1.04,10.74)**	**4.55 (1.45,14.34)**	**4.70 (1.40,15.81)**	5.07 (1.74,14.80)	**4.00 (1.37,11.68)**	2.36 (0.80,6.95)	**7.16 (1.96,26.12)**	**5.81 (1.92,17.52)**	2.62 (0.85,8.08)	Betaine	0.38 (0.14, 1.04)
0.94 (0.53, 1.66)	1.26 (0.71, 2.24)	**1.72 (1.01, 2.93)**	1.78 (0.91, 3.44)	**1.91 (1.36, 2.69)**	**1.51 (1.07, 2.13)**	0.89 (0.61, 1.29)	**2.7 (1.21, 6.02)**	**2.19 (1.42, 3.38)**	0.99 (0.61, 1.61)	0.38 (0.14, 1.04)	Placebo

Appendix Table 5. Pairwise (direct) estimates effects of different interventions compared with comparators (co-intervention: advise of weight and diet control) on mean difference of histological outcomes (outcomes reported as Weighted Mean Difference (WMD) along with 95%CI in parenthesis)

**Intervention**	**N (study)**	**Fibrosis**	**GRADE**	**N (study)**	**NAS**	**GRADE**	**n (study)**	**Inflammation**	**GRADE**	**n (study)**	**Ballooning**	**GRADE**	**n (study)**	**Steatosis**	**GRADE**
**Placebo control**															
PTX	85 (2)	**-0.60 (-0.95, -0.25)**	⊕⊕⊕⊕ HIGH	108 (3)	**-1.50 (-1.87, -1.14)**	⊕⊕⊕⊕ HIGH	108 (3)	**-0.50 (-0.72, -0.27)**	⊕⊕⊕⊕ HIGH	148 (4)	-0.31 (-0.76, 0.14)	⊕◯◯◯ VERY LOW[1,2,3]	N/A	N/A	N/A
OCA	283 (1)	**-0.30 (-0.52, -0.08)**	⊕⊕⊕⊕ HIGH	283 (1)	**-1.00 (-1.42, -0.58)**	⊕⊕⊕⊕ HIGH	283 (1)	**-0.30 (-0.50, -0.10)**	⊕⊕⊕⊕ HIGH	283 (1)	**-0.30 (-0.51, -0.09)**	⊕⊕⊕⊕ HIGH	283 (1)	**-0.40 (-0.61, -0.19)**	⊕⊕⊕⊕ HIGH
Betaine	55 (1)	-0.20 (-0.88, 0.48)	⊕⊕⊕◯ MODERATE1	55 (1)	0.80 (-0.36, 1.96)	⊕⊕⊕◯ MODERATE[1]	55 (1)	-0.16 (-0.72, 0.40)	⊕⊕⊕◯ MODERATE[1]	55 (1)	0.35 (-0.08, 0.78)	⊕⊕⊕◯ MODERATE[1]	**55 (1)**	**-1.10 (-1.63, -0.57)**	⊕⊕⊕⊕ HIGH
BIB	21 (1)	0.50 (-0.46, 1.46)	⊕⊕⊕◯ MODERATE[1]	21 (1)	**-2.00 (-3.11, -0.89)**	⊕⊕⊕⊕ HIGH	21 (1)	NA	N/A	21 (1)	NA	N/A	N/A	N/A	N/A
Vitamin E	116 (1)	-0.10 (-1.87, 1.67)	⊕⊕⊕◯ MODERATE[1]	116 (1)	**-1.10 (-1.15, -1.05)**	⊕⊕⊕⊕ HIGH	116 (1)	-0.10 (-1.33, 1.33)	⊕⊕⊕◯ MODERATE[1]	116 (1)	-0.60 (-1.96, 0.76)	⊕⊕⊕◯ MODERATE[1]	116 (1)	-0.40 (-2.17, 1.37)	⊕⊕⊕◯ MODERATE[1]
Antiox	161 (2)	-0.09 (-0.18, 0.01)	⊕⊕◯◯ LOW [1,2]	161 (2)	-0.69 (-2.03, 0.65)	⊕⊕◯◯ LOW [1,2]	60 (1)	-0.20 (-0.50, 0.10)	⊕⊕◯◯ LOW [1,2]	60 (1)	-0.27 (-0.58, 0.04)	⊕⊕◯◯ LOW [1,2]	**60 (1)**	**-0.92 (-1.33, -0.51)**	⊕⊕⊕◯ MODERATE[2]
PUFA	340 (3)	-0.04 (-0.38, 0.29)	⊕⊕⊕◯ MODERATE[1]	303 (2)	0.04 (-0.32, 0.39)	⊕⊕⊕◯ MODERATE[1]	340 (3)	-0.01 (-0.41, 0.38)	⊕⊕◯◯ LOW [1,3]	240 (3)	-0.05 (-0.31, 0.20)	⊕⊕⊕◯ MODERATE[1]	N/A	N/A	N/A
Met	236 (5)	0.41 (0.14, 0.68)	⊕⊕⊕◯ MODERATE[2]	231 (4)	0.53 (-0.40, 1.47)	⊕◯◯◯ VERY LOW[1,2,3]	183 (3)	0.47 (0.15, 0.79)	⊕⊕⊕◯ MODERATE[2]	183 (3)	0.22 (-0.18, 0.61)	⊕◯◯◯ VERY LOW[1,2,3]	183 (3)	**-0.47 (-0.79, -0.15)**	⊕⊕⊕◯ MODERATE[2]
TZD	167 (3)	0.40 (-0.99, 1.79)	⊕◯◯◯ VERY LOW [1,2,3]	113 (2)	0.03 (-0.29, 0.35)	⊕⊕◯◯ LOW [1,2]	167 (3)	-0.00 (-0.65, 0.64)	⊕◯◯◯ VERY LOW[1,2,3]	**207 (4)**	**-0.31 (-0.56, -0.07)**	⊕⊕◯◯ LOW [2,3]	**104 (2)**	**-0.66 (-0.82, -0.50)**	⊕⊕⊕◯ MODERATE[2]
Weight and lipid control	237 (5)	0.20 (-0.26, 0.66)	⊕⊕◯◯ LOW [1,2]	**163 (4)**	**-0.87 (-1.44, -0.30)**	⊕⊕⊕◯ MODERATE[2]	237 (5)	0.20 (-0.26, 0.66)	⊕◯◯◯ VERY LOW[1,2,3]	237 (5)	-0.22 (-0.71, 0.27)	⊕◯◯◯ VERY LOW[1,2,3]	253 (6)	-0.38 (-0.95, 0.20)	⊕◯◯◯ VERY LOW [1,2,3]
UDCA	477 (4)	**0.14 (0.07, 0.21)**	⊕⊕⊕⊕ HIGH	178 (2)	-0.02 (-0.26, 0.30)	⊕⊕◯◯ LOW [1,3]	384 (3)	-0.16 (-0.48, 0.17)	⊕⊕◯◯ LOW [1,3]	348 (3)	0.09 (-0.03, 0.21)	⊕⊕⊕◯ MODERATE[1]	348 (3)	-0.03 (-0.17, 0.10)	⊕⊕⊕◯ MODERATE[1]
Metadoxine	134 (1)	0.02 (-0.26, 0.30)	⊕⊕⊕◯ MODERATE[1]	N/A	N/A	N/A	N/A	N/A	N/A	N/A	N/A	N/A	134 (1)	**-0.45 (-0.77, -0.13)**	⊕⊕⊕⊕ HIGH
Antiox + UDCA	100 (1)	**-0.29 (-0.35, -0.23)**	⊕⊕⊕⊕ HIGH	100 (1)	**-0.34 (-0.40, -0.28)**	⊕⊕⊕⊕ HIGH	N/A	N/A	N/A	N/A	N/A	N/A	N/A	N/A	N/A

Vitamin E + Vitamin C	139 (2)	-0.09 (-0.29, 0.11)	⊕⊕◯◯ LOW [1][2]	90 (1)	0.00 (-0.49, 0.49)	⊕⊕◯◯ LOW [1][2]	90 (1)	-0.10 (-0.31, 0.11)	⊕⊕◯◯ LOW [1][2]	90 (1)	-0.20 (-0.49, 0.09)	⊕⊕◯◯ LOW [1][2]	90 (1)	**-0.50 (-0.86, -0.14)**	⊕⊕⊕◯ MODERATE[2]
Vitamin E + UDCA	30 (1)	-0.90 (-1.82, 0.02)	⊕⊕⊕◯ MODERATE[1]	N/A	N/A	N/A	30 (1)	-0.50 (-1.15, 0.15)	⊕⊕⊕◯ MODERATE[1]	30 (1)	-0.40 (-1.38, 0,58)	⊕⊕⊕◯ MODERATE[1]	30 (1)	-0.80 (-1.67, 0.07)	⊕⊕⊕◯ MODERATE[1]
**Interventions (vs. active controls)**															
Bicyclol (vs. Vitamin E)	248 (1)	-0.12 (-0.32, 0.08)	⊕⊕◯◯ LOW [1][2]	N/A	N/A	N/A	248 (1)	**-0.65 (-0.80, -0.50)**	⊕⊕⊕◯ MODERATE[2]	248 (1)	0.10 (-0.03, 0.23)	⊕⊕◯◯ LOW [1][2]	248 (1)	**-0.21 (-0.37, -0.05)**	⊕⊕⊕◯ MODERATE[2]
Met (vs. Vitamin E)	115 (1)	-0.10 (-1.87, 1.67)	⊕⊕⊕◯ MODERATE[1]	115 (1)	0.70 (0.64, 0.76)	⊕⊕⊕⊕ HIGH	115 (1)	0.10 (-1.13, 1.33)	⊕⊕⊕◯ MODERATE[1]	115 (1)	0.20 (-1.30, 1.70)	⊕⊕⊕◯ MODERATE[1]	115 (1)	0.20 (-1.57, 1.97)	⊕⊕⊕◯ MODERATE[1]
Met (vs. TZD)	49 (1)	-1.00 (-1.24, -0.76)	⊕⊕⊕◯ MODERATE[2]	91 (2)	1.95 (-0.27, 4.17)	⊕⊕◯◯ LOW [1][2]	49 (1)	0.00 (-0.33, 0.33)	⊕⊕◯◯ LOW [1][2]	49 (1)	1.00 (0.86, 1.14)	⊕⊕⊕◯ MODERATE[2]	49 (1)	0.00 (-0.33, 0.33)	⊕⊕⊕◯ MODERATE[2]
Antiox (vs. UDCA)	**100 (1)**	**-0.23 (-0.31, -0.15)**	⊕⊕⊕⊕ HIGH	**100 (1)**	**-0.14 (-0.24, -0.04)**	⊕⊕⊕⊕ HIGH	N/A	N/A	N/A	N/A	N/A	N/A	N/A	N/A	N/A
Antiox + UDCA (vs. UDCA)	**99 (1)**	**-0.46 (-0.52, -0.40)**	⊕⊕⊕⊕ HIGH	**100 (1)**	**-0.46 (-0.52, -0.40)**	⊕⊕⊕⊕ HIGH	N/A	N/A	N/A	N/A	N/A	N/A	N/A	N/A	N/A
Vitamin E +UDCA (vs. UDCA)	33 (1)	-0.70 (-1.69, 0.29)	⊕⊕⊕◯ MODERATE[1]	N/A	N/A	N/A	33 (1)	-0.20 (-0.79, 0.39)	⊕⊕⊕◯ MODERATE[1]	33 (1)	-0.20 (-1.04, 0.64)	⊕⊕⊕◯ MODERATE[1]	33 (1)	**-0.90 (-1.75, -0.05)**	⊕⊕⊕⊕ HIGH
TZD + Vitamin E (vs. Vitamin E)	N/A	N/A	N/A	N/A	N/A	N/A	N/A	N/A	N/A	20 (1)	-0.30 (-1.54, 0.94)	⊕⊕◯◯ LOW [1][2]	20 (1)	-0.50 (-2.95, 1.95)	⊕⊕◯◯ LOW [1][2]
TZD + Met (vs. Met)	44 (1)	-4.60 (-7.12,-2.08)	⊕⊕⊕◯ MODERATE[1]	N/A	N/A	N/A	N/A	N/A	N/A	N/A	N/A	N/A	N/A	N/A	N/A
TZD + Met (vs. TZD)	42 (1)	-1.30 (-3.77, 1.17)	⊕⊕⊕◯ MODERATE[1]	N/A	N/A	N/A	N/A	N/A	N/A	N/A	N/A	N/A	N/A	N/A	N/A
TZD (vs. PTX)	60 (1)	-0,50 (-0.77, -0.23)	⊕⊕⊕⊕ HIGH	N/A	N/A	N/A	N/A	N/A	N/A	60 (1)	-0.19 (-0.51, 0.13)	⊕⊕⊕◯ MODERATE[1]	N/A	N/A	N/A

Appendix Table 6. League table presenting network meta-analysis estimates (lower triangle) and direct estimates (upper triangle) for change of fibrosis grade

Vitamin E+ Vitamin C	-	-	-	-	-	-	-	-	-	-	-	-	-	-	-	-0.09 (-0.29, 0.11)
0.80 (-0.18, 1.78)	Vitamin E+ UDCA	-	-	-0.70 (-1.69, 0.29)	-	-	-	-	-	-	-	-	-	-	-	-0.90 (-1.82, 0.02)
0.21 (-0.16, 0.57)	-0.61 (-1.58, 0.36)	Antiox + UDCA	-	-0.46 (-0.52, -0.40)	-	-	-	-	-	-	-	-	-	-	-	-0.29 (-0.35, -0.23)
-0.05 (-1.88, 1.78)	-0.78 (-2.85, 1.29)	-0.35 (-2.22, 1.51)	Vitamin E	-	-	-	-	-	-	-	-0.10 (-1.87, 1.67)	-0.12 (-0.32, 0.08)	-	-	-	-0.10 (-1.87, 1.67)
-0.25 (-0.62, 0.11)	-0.70 (-1.71, 0.31)	-0.46 (-0.69, -0.23)	-0.27 (-2.07, 1.53)	UDCA	-	-	-	-	-	-	-	-	-	-	-0.23 (-0.31, -0.15)	**0.14 (0.07, 0.21)**
0.19 (-0.26, 0.65)	-0.62 (-1.64, 0.40)	-0.01 (-0.45, 0.43)	0.18 (-1.64, 2.00)	0.45 (0.005, 0.89)	Weight or lipid control	-	-	-	-	-	-	-	-	-	-	0.20 (-0.26, 0.66)
**-1.58 (-2.05, -1.10)**	**-2.40 (-3.42, -1.38)**	**-1.78 (-2.23, -1.34)**	-1.59 (-3.42, 0.23)	**-1.32 (-1.77, -0.88)**	**-1.77 (-2.31, -1.23)**	TZD	-	-0.50 (-0.77, -0.23)	-	-	-1.00 (-1.24, -0.76)	-	-	-	-	0.40 (-0.99, 1.79)
-0.07 (-0.42, 0.29)	-0.88 (-1.85, 0.08)	-0.28 (-0.59, 0.04)	-0.08 (-1.88, 1.71)	0.18 (-0.123, 0.50)	-0.26 (-0.71, 1,84)	1.51 (1.08, 1.95)	PUFA	-	-	-	-	-	-	-	-	-0.04 (-0.38, 0.29)
0.51 (0.03, 0.10)	-0.30 (-1.32, 0.72)	0.31 (-0.14, 0.76)	0.50 (-1.33, 2.32)	0.77 (0.31, 1.22)	0.32 (-0.22, 0.86)	2.10 (1.55, 2.64)	0.58 (0.14, 1.02)	PTX	-	-	-	-	-	-	-	**-0.60 (-0.95, -0.25)**
0.22 (-0.20, 0.64)	-0.60 (-1.60, 0.40)	0.009 (-0.38, 0.40)	0.20 (-1.61, 2.01)	0.47 (0.08, 0.86)	0.02 (-0.47, 0.51)	1.80 (1.31, 2.29)	0.28 (-0.09, 0.66)	-0.30 (-0.80, 0.20)	OCA	-	-	-	-	-	-	**-0.30 (-0.52, -0.08)**
-0.10 (-0.56, 0.35)	-0.92 (-1.93, 0.09)	-0.31 (-0.73, 0.11)	-0.12 (-1.94, 1.70)	0.15 (-0.27, 0.57)	-0.30 (-0.82, 0.22)	1.48 (0.96, 1.99)	-0.03 (-0.44, 0.38)	-0.62 (-1.15, -0.09)	-0.32 (-0.79, 0.15)	Metadoxine	-	-	-	-	-	0.02 (-0.26, 0.30)
-0.25 (-0.89, 0.39)	-1.06 (-2.17, 0.04)	-0.46 (-1.08, 0.16)	0.10 (-1.68, 1.88)	0.004 (-0.62, 0.63)	-0.44 (-1.13, 0.39)	1.00 (0.67, 1.33)	-0.18 (-0.80, 0.43)	-0.76 (-1.46, -0.07)	-0.46 (-1.12, 0.19)	-0.14 (-0.82, 0.53)	Met	-	-	-	-	**0.41 (0.14, 0.68)**
0.12 (-1.64, 1.89)	-0.69 (-2.67, 1.29)	-0.08 (-1.84, 1.68)	0.12 (-0.18, 0.42)	0.26 (-1.49, 2.02)	-0.07 (-1.85, 1.71)	-0.16 (-1.94, 1.62)	0.19 (-1.56, 1.95)	-0.39 (-2.17, 1.40)	-0.09 (-1.86, 1.68)	0.23 (-1.55, 2.00)	0.02 (-1.78, 1.81)	Bicyclol	-	-	-	-
0.11 (-0.65, 0.88)	-0.70 (-1.89, 0.48)	-0.09 (-0.84, 0.66)	0.10 (-1.82, 2.01)	0.37 (-0.38, 1.12)	-0.8 (-0.89, 0.72)	1.70 (0.89, 2.50)	0.18 (-0.56, 0.93)	-0.40 (-1.21, 0.41)	-0.10 (-0.88, 0.68)	0.22 (-0.58, 1.01)	0.36 (-0.55, 1.28)	-0.02 (-1.96, 1.92)	Betaine	-	-	-0.20 (-0.88, 0.48)
-0.57 (-1.59, 0.44)	-1.39 (-2.75, -0.03)	-0.78 (-1.78, 0.22)	-0.59 (-2.62, 1.44)	-0.32 (-1.32, 0.68)	-0.77 (-1.81, 0.28)	1.01 (-0.03, 2.06)	-0.50 (-1.50, 0.49)	-1.09 (-2.14, -0.04)	-0.79 (-1.81, 0.24)	-0.47 (-1.50, 0.57)	-0.32 (-1.45, 0.81)	-0.71 (-2.76, 1.35)	-0.69 (-1.90, 0.52)	BIB	-	0.50 (-0.46, 1.46)
0.09 (-0.31, 0.49)	-0.73 (-1.72, 0.26)	-0.23 (-0.46, 0.005)	0.07 (-1.74, 0.26)	0.23 (-0.008, 0.47)	-0.11 (-0.58, 0.36)	1.67 (1.19, 2.14)	0.15 (-0.20,0.51)	-0.43 (-0.91, 0.05)	-0.13 (-0.55, 0.29)	0.19 (-0.26, 0.64)	0.34 (-0.31, 0.98)	-0.05 (-1.88, 1.78)	-0.03 (-0.80, 0.74)	0.67 (-0.36, 1.70)	Antiox	-0.09 (-0.18, 0.01)
-0.08 (-0.36, 0.20)	-0.90 (-1.85, 0.06)	-0.29 (-0.52, -0.06)	-0.10 (-1.88, 1.68)	0.17 (-0.06, 0.40)	-0.28 (-0.66, 0.10)	1.50 (1.12, 1.88)	-0.02 (-0.22, 0.20)	-0.60 (-0.99, -0.21)	-0.30 (-0.61, 0.01)	0.02 (-0.33, 0.37)	0.17 (-0.41, 0.75)	-0.22 (-2.03, 1.59)	-0.20 (-0.92, 0.52)	0.50 (-0.48, 1.48)	-0.06 (-0.30, 0.18)	Placebo

Appendix Table 7. Assessing inconsistency tests of network meta-analysis at network levels

**Outcomes**	**Likelihood ratio test**	**Inconsistency tests of binary outcomes**		**Likelihood ratio test**	**Inconsistency tests of continuous outcomes**	
		X^2	***p*-value**		X^2	***p*-value**
**Fibrosis**	-14.69	4.17	0.3829	-16.52	74.62	0.0000
**Resolution of NASH**	-4.96	0.18	0.6716	N/A	N/A	N/A
**NAS**	-7.59	6.66	0.0837	-14.96	89.33	0.0000
**Steatosis**	-12.13	4.21	0.2397	-22.71	0.93	0.9681
**Ballooning degeneration**	-10.59	2.64	0.4503	-19.09	3.00	0.7008
**Lobular inflammation**	-9.94	6.05	0.1090	-15.44	6.96	0.2234

Appendix Table 8. Sensitivity and subgroup analyzes of network meta-analysis for improvement of fibrosis outcome

Interventions	**Estimated effects of network meta-analysis; Risk ratio, RR (95%CI)**				
	Main analysis	Small studies omitted	Low quality studies omitted	Included only studies with F/U ≥1 year	Included only studies with F/U < 1 year
PTX	2.27 (0.81, 6.36)	N/A	2.30 (0.78, 6.71)	5.91 (0.37, 95.02)	1.95 (0.64, 5.91)
**Obeticholic acid**	**1.91 (1.15, 3.16)**	**1.91 (1.15, 3.16)**	**1.91 (1.04, 3.50)**	**1.91 (1.15, 3.16)**	N/A
Weight or lipid control	1.74 (0.55, 5.51)	N/A	1.74 (0.53, 5.69)	N/A	2 (0.68, 5.85)
TZD + Met	1.52 (0.79, 2.94)	1.5 (0.77, 2.95)	N/A	1.5 (0.77, 2.94)	N/A
Metadoxine	1.35 (0.57, 3.21)	1.35 (0.57, 3.21)	1.35 (0.53, 3.42)	N/A	1.35 (0.57, 3.21)
TZD	1.33 (0.96, 1.83)	1.31 (0.92, 1.87)	1.28 (0.76, 2.15)	1.31 (0.92, 1.87)	1.48 (0.69, 3.17)
Vitamin E + Vitamin C	1.27 (0.66, 2.44)	3 (0.32, 27.76)	1.17 (0.55, 5.69)	3 (0.32, 27.76)	1.17 (0.59, 2.32)
Met	1.25 (0.81, 1.93)	1.34 (0.85, 2.1)	1.20 (0.67, 2.16)	1.34 (0.85, 2.10)	0.34 (0.05, 2.20)
TZD + Losartan	1.24 (0.61, 2.53)	1.23 (0.59, 2.54)	N/A	1.23 (0.59, 2.54)	N/A
Vitamin E	1.23 (0.89, 1.69)	1.24 (0.89, 1.72)	1.18 (0.76, 1.83)	1.24 (0.89, 1.72)	N/A
UDCA	0.90 (0.41, 1.96)	0.9 (0.41, 1.96)	0.90 (0.38, 2.10)	0.90 (0.41, 1.96)	N/A
PUFA	0.58 (0.22, 1.57)	N/A	0.58 (0.21, 1.62)	N/A	N/A
Betaine	N/A	N/A	N/A	0.58 (0.22, 1.57)	N/A

Appendix Table 9. Ranking of interventions in network meta-analysis for binary outcomes, after included studies with sample sizes exceed 25th percentile

Interventions	Risk Ratio: RR (95%CI)	pbest (%)	Rank	SUCRA	Rank
Fibrosis					
Vitamin E + Vitamin C	3 (0.32, 27.76)	32.5	2	0.7	2
**OCA**	**1.91 (1.15, 3.16)**	6.5	4	0.7	2
TZD + Met	1.5 (0.77, 2.95)	39.5	1	0.8	1
Metadoxine	1.35 (0.57, 3.21)	2.8	5	0.5	3
Met	1.34 (0.85, 2.1)	0.4	7	0.4	4
TZD	1.31 (0.92, 1.87)	1.4	6	0.4	4
Vitamin E	1.24 (0.89, 1.72)	0.1	9	0.2	5
TZD + LOS	1.23 (0.59, 2.54)	16.6	3	0.7	2
UDCA	0.9 (0.41, 1.96)	0.3	8	0.2	5
Resolution NASH					
**TZD**	**2.42 (1.53, 3.82)**	0.367	1	0.8	1
**Vitamin E**	**2.07 (1.39, 3.09)**	0.297	2	0.8	1
LOS + TZD	1.83 (0.78, 4.32)	0.113	4	0.5	2
OCA	1.70 (0.89, 3.25)	0.076	5	0.4	3
Met + TZD	1.68 (0.71, 3.99)	0.021	6	0.4	3
Met	1.44 (0.82, 2.52)	0.126	3	0.5	2
NAS					
**Vitamin E**	**2.24 (1.52, 3.31)**	0.403	1	0.8	1
**OCA**	**2.19 (1.42, 3.38)**	0.302	2	0.8	1
LOS + TZD	(1.07, 3.76)2	0.229	3	0.8	1
TZD	1.76 (1.09, 2.84)	0.035	4	0.6	2
Met	1.47 (0.83, 2.60)	0.016	5	0.5	3
Met + TZD	1.47 (0.76, 2.86)	0.015	6	0.5	3
Vitamin E + Vitamin C	0.94 (0.53, 1.66)	0.001	7	0.2	4
PUFA	0.87 (0.56, 1.35)	0	8	0.1	5
Steatosis					
**MDX**	**2.97 (1.55, 5.70)**	0.427	1	0.8	1
**LOS + TZD**	**2.78 (1.61, 4.78)**	0.28	2	0.8	1
**Met + TZD**	**2.35 (1.34, 4.12)**	0.133	3	0.7	2
**TZD**	**1.95 (1.47, 2.59)**	0.02	6	0.6	3
**OCA**	**1.69 (1.21, 2.36)**	0.061	4	0.5	4
**Vitamin E**	**1.64 (1.23, 2.18)**	0.028	5	0.4	5
**Met**	**1.53 (1.02, 2.28)**	0.028	5	0.4	5
UDCA	1.18 (0.71, 1.95)	0.017	7	0.3	6
Vitamin E + Vitamin C	1.00 (0.56, 1.79)	0.008	8	0.2	7
Ballooning					
**Met**	**2.13 (1.28, 3.54)**	0.36	1	0.8	1
**Vitamin E**	**2.06 (1.45, 2.93)**	0.191	2	0.8	1
**TZD**	**1.72 (1.15, 2.58)**	0.024	7	0.6	2
TZD + LOS	1.71 (0.76, 3.84)	0.182	**3**	0.6	2
OCA	1.58 (1.06, 2.34)	0.055	6	0.5	3
Met + TZD	1.44 (0.63, 3.3)	0.074	5	0.5	3
UDCA	1.34 (0.56, 3.23)	0.11	4	0.4	4

Interventions	Risk Ratio: RR (95%CI)	pbest (%)	Rank	SUCRA	Rank
Vitamin E + Vitamin C	0.50 (0.16, 1.54)	0.004	8	0	5
Lobular inflammation					
**OCA**	**1.60 (1.05, 2.43)**	0.259	1	0.8	1
**TZD**	**1.59 (1.11, 2.17)**	0.283	2	0.8	1
LOS + TZD	1.45 (0.65, 3.21)	0.247	3	0.7	2
**Vitamin E**	**1.44 (1.03, 2.01)**	0.022	7	0.4	4
Met	1.35 (0.84, 2.17)	0.036	6	0.5	3
Met + TZD	1.22 (0.54, 2.76)	0.101	4	0.5	3
Vitamin E + Vitamin C	1.00 (0.49, 2.05)	0.039	5	0.3	5
UDCA	0.74 (0.33, 1.69)	0.013	8	0.2	6
